# Bidirectional associations between sensorineural hearing loss and depression and anxiety: a meta-analysis

**DOI:** 10.3389/fpubh.2023.1281689

**Published:** 2024-01-08

**Authors:** Zhi-qiang Zhang, Jing-yang Li, Si-tong Ge, Tian-yi Ma, Fu-yao Li, Jun-liang Lu, Shu-rui Si, Zhe-zhu Cui, Yu-lian Jin, Xiang-hua Jin

**Affiliations:** ^1^Department of Otolaryngology, Head and Neck Surgery, Affiliated Hospital of Yanbian University, Yanji, China; ^2^Department of Clinical Medicine, First School of Clinical Medicine, Nanchang University, Nanchang, China; ^3^Department of Psychiatry, Yanbian University Hospital, Yanji, China; ^4^Department of Otorhinolaryngology, Head and Neck Surgery, Xinhua Hospital, Shanghai Jiaotong University School of Medicine, Shanghai, China; ^5^Shanghai Jiaotong University School of Medicine Ear Institute, Shanghai, China; ^6^Shanghai Key Laboratory of Translational Medicine on Ear and Nose Diseases, Shanghai, China

**Keywords:** sensorineural hearing loss, anxiety, depression, meta-analysis, mental disorder

## Abstract

**Background:**

Recently, the prevalence of sensorineural hearing loss (SNL) has been increasing, and several studies have suggested that depression, anxiety, and SNL may be associated with each other, however, individual findings still have discrepancies. To the best of our knowledge, no scholars have systematically elucidated the bidirectional associations between SNL, depression, and anxiety disorders from the perspective of meta-analysis. In this study, we aimed to systematically evaluate the bidirectional associations between SHL and depressive and anxiety symptoms, and to provide evidence-based medical evidence for reducing SNL, depression, and anxiety disorders.

**Methods:**

We performed systematic review based on priori protocol that was registered with PROSPERO (No. CRD42022365963). Systematic search of PubMed, Embase, and Web of Science databases identified articles published as of June 1, 2023, on the relationship between SNL and depression and anxiety. Meta-analysis was performed to calculate the odds ratios (OR) and 95% confidence intervals (CIs) for the outcome metrics, and the results were combined to assess bivariate associations between the disorders with fixed or random effects. Sensitivity and subgroup analyzes were conducted to analyze sources of heterogeneity, and Egger’s and Begg’s tests combined with funnel plots were applied to assess publication bias.

**Results:**

Summary analysis of the results of 20 studies covering 675,291 individuals showed that the bidirectional association between SNL and depression and anxiety disorders. The incidence (OR = 0.17, 95% CI: 0.09–0.28) and risk (OR = 1.43, 95% CI: 1.32–1.55) of depression and morbidity were higher in SNL patients than the general population. Elevated prevalence (OR = 0.46, 95% CI: 0.28–0.65) and risk (OR = 1.30, 95% CI: 1.11–1.48) of SNL were also observed in depressed patients. The prevalence of anxiety disorders among SNL patients was about 40% (OR = 0.40, 95% CI: 0.24%-0.57), which was associated with higher risk (OR = 1.83, 95% CI: 1.42–2.24) of development than the general population. Incidence of SNL in patients with anxiety disorders was approximately 31% (OR = 0.31, 95% CI: 0.29–0.33). Additionally, subgroup analyzes showed that the bidirectional associations between SNL, depression, and anxiety disorders was influenced by age, region, and mode of diagnosis of the disorders (SNL, depression, anxiety).

**Conclusion:**

There are bidirectional associations between SNL and depression and anxiety disorders, which was influenced by age and region and the method the disorders (SNL, depression, anxiety) were diagnosed.

## 1 Introduction

Recently, the incidence of sensorineural hearing loss (SHL) has been increasing year by year, with more than 1.5 billion people around the world suffering from varying degrees of SHL, including at least 430 million people suffering from moderate SHL or worse (1). Notably, the prevalence of SHL may increase and become worse with age, studies have shown that the frequency of SHL is approximately four times greater in older adults aged 90 and over than in 60-year-olds, which is accompanied by more severe SHL (2).

Over the past two decades, depression and anxiety disorders have become one of the major public health concerns globally and are considered to be the most common mental disorders, affecting more than 264 million people worldwide and potentially leading to severe mental stress and dysfunction, and even suicide, especially in low- and middle-income countries (3). Furthermore, depression and anxiety increased in prevalence with age (4), with approximately 15% of older adults experiencing clinically significant depressive symptoms and 1–5% suffering from major anxiety disorders (5).

Various studies (6–11) have shown that SHL may contribute to the more frequent occurrence of depression and anxiety disorders. Interestingly, depression and anxiety could also be responsible for the development and progression of SHL (9). Associations have been reported between SHL and depression and anxiety disorders in recent years (7, 10, 12–14), nevertheless, the majority of studies have focused merely on the effect of SHL on the risk of depression and anxiety disorders (6–8, 10, 15, 16), while evidence of an inverse association between SHL and events of depression and anxiety disorders is limited, and the results of the various studies have been inconsistent and the conclusions are still somewhat controversial. To the best of our knowledge, currently nobody has systematically elaborated the bidirectional associations between SHL and depression and anxiety disorders from the perspective of evidence-based medicine, therefore the systematic evaluation and meta-analysis of the existing evidence is necessitated.

The primary objective of this meta-analysis was to succinctly summarize the existing evidence on the prevalence of depression and anxiety disorders in SHL. Additionally, it aimed to evaluate the bidirectional associations related to the risk of developing depression and anxiety disorders in individuals with SHL. The ultimate goal is to provide valuable insights for clinical practitioners.

## 2 Methods

### 2.1 Protocol and registration

This study is reported according to the Preferred Reporting Items for Systematic Reviews and Meta-Analysis (PRISMA) (17). We performed a systematic review based on *a priori* protocol that was registered with PROSPERO (No. CRD42022365963), which was to ensure the originality of our selected topic.

### 2.2 Eligibility criteria

Inclusion criteria: (1) patients with SHL whose exposure was confirmed by pure tone audiometry (PTA), questionnaires, or self-reported hearing loss; (2) patients with or at risk for outcome-confirmed diagnosis or self-reported depression or anxiety via the Depression or Anxiety Scale; (3) age of the patient needs to be ≥18 years old for either the exposure or the outcome; and (4) type of study: observational, which can be a case–control study, cohort study, or cross-sectional study.

Exclusion criteria included: Literature with no access to full text and missing raw data; Literature with illogical study design protocols; Literature that did not report on the ethical review process; and meeting abstracts, reviews and Letters were also excluded.

### 2.3 Search strategy

The computerized search of PubMed, Embase, and Web of Science databases was conducted to assemble case–control studies, cohort studies, or cross-sectional studies on the relationship between SHL and depression and anxiety. All search timeframes were from library construction to June 1, 2023. The English search terms included Hypoacusis, Hearing Loss, Hypoacusis, Hearing Impairment, Deafness, Depression, Depressive Symptom, Emotional Depression, Melancholia, Anxiety, Angst, Hypervigilance, Nervousness, Anxiousness, etc. Detailed literature search formula and search details are shown in Supplementary Table S1.

### 2.4 Data extraction and quality assessment

Literature was screened, extracted and cross-checked independently by 2 researchers, consulting a third party for assistance in any disagreements, and contacting the corresponding authors to supplement any missing information wherever possible. During literature screening, the title and abstract were read initially, and obviously irrelevant literature was excluded, and then the full text was read further to determine final inclusion. The extracted data mainly included: basic information of the included studies, including the first author, investigation area, publication time, etc.; sample size of the study population, patients’ (average) age, gender and disease diagnosis criteria; outcome indicators and result measures (prevalence rate, ratio, risk ratio, etc.) of the studies; specific details of the interventions, disease status after the interventions, etc.; and the key elements of the evaluation of the risk of bias. Two investigators independently evaluated the risk of bias of the included studies, and any disagreement was resolved through discussion and negotiation. Risk of bias was assessed using the New Castle-Ottawa scale (NOS) for case–control and cohort studies, and the Agency for Healthcare Research and Quality (AHRQ) risk of bias criteria for cross-sectional studies, and ≥ 5 were classified as high quality.

### 2.5 Statistical analysis

Stata 16.0 software were used to perform this meta-analysis. Measurement data utilized the odds ratio (OR) and the combined percentage as effect indicators, each of which was provided with points estimates and 95% confidence intervals (CIs). Heterogeneity between the results of the included studies was analyzed using the *X*^2^ test (the test level was *α* = 0.1), and the magnitude of heterogeneity was quantitatively determined by combining with *I*^2^. Fixed effects model was used to combine the effects if *I*^2^ was ≤50%, and random effects model to combine the effects if *I*^2^ was >50%, followed by sensitivity analysis or subgroup analysis to explore the source of heterogeneity. Funnel plots were drawn for outcome metrics for ≥6 articles included in the literature and combined with Begg’s and Egger’s tests to assess publication bias.

## 3 Result

### 3.1 Retrieved literatures and study characteristics

**Figure 1:**
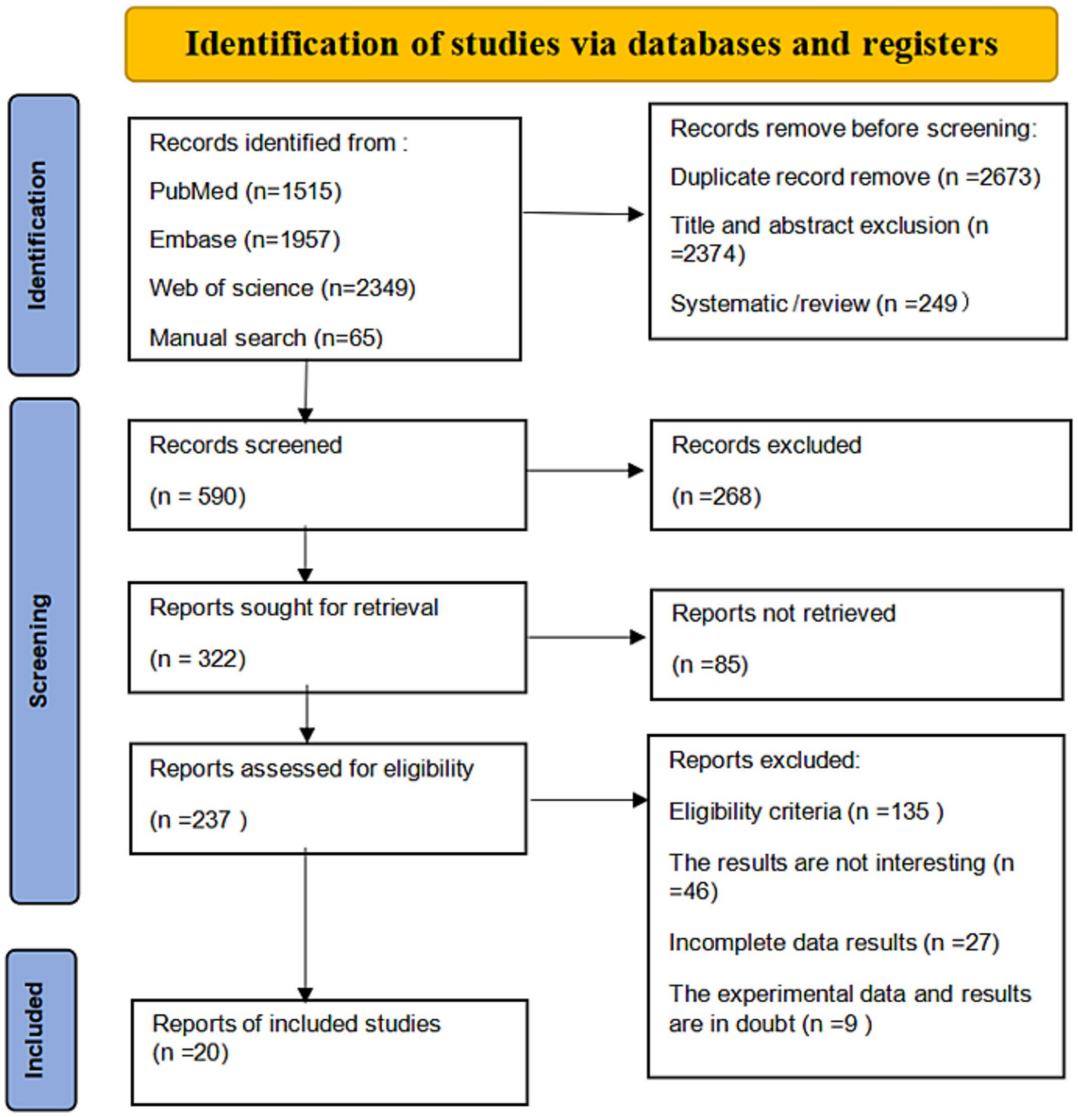
Literature screening flowchart.

Five thousand, eight hundred eighty-six articles were initially generated from database and manual searches. Critically reviewed based on title and abstract by two independent reviewers after removing duplicate studies. 236 papers were selected for evaluation in full text and finally, according to the previously established inclusion criteria, 20 articles were incorporated into this study for the meta-analysis. The detailed literature screening process is presented in Figure 1. Ultimately, 20 studies containing 675,291 subjects participated in this meta-analysis, and the major characteristics of the included studies are shown in Table 1. Among all eligible studies included, 7 are cross-sectional, while 6 are case–control and 7 are cohort studies.

**Table 1:** Basic characteristics of the included literature.

Author	Year	Country	Type	Diagnostic criteria (SNL)	Diagnostic criteria (Depression/Anxiety)	Sample	Gender (man/woman)	Age	Outcome
Tseng et al. (6)	2016	Taiwan	C-C	PTA	MD	8,585	4730/3855	39–63 (IQR)	①④⑤
Kim et al. (7)	2018	Korea	C-C	PTA	MD	8,550	4147/4403	50 (median)	①②③ ④⑤⑥⑦
Hsu et al. (8)	2016	Taiwanese	CS	HIS	DDS(CESD-10)/ADS(ICD-9-CM)	25,215	15,605/9610	61.8 ± 17.0	①②④⑤
Wu et al. (9)	2021	China	C-C	SR	DDS(CESD-10)	8,339	-	58.1 ± 9.0	①③
Kim et al. (10)	2020	Korea	CS	PTA	MD	325,230	113,405/211825	55.5 ± 8.4	①③
Chung et al. (11)	2015	Taiwan	C-C	PTA	MD	14,088	-	51.3	②⑥⑦
Cejas et al. (12)	2021	United States	C-S	PTA	Depression SR(PHQ-8) ADS(GAD-7)	104	-	12–18	④⑥
Yang et al. (13)	2021	China	C-S	HIS	DDS(DASS-21)/ADS(DASS-21)	706	280/426	13–27	④
Powell et al. (14)	2021	United States	CS	PTA	DDS (CES-D)	2061	989/1072	74 ± 2.8	④
Kim et al. (15)	2017	Korea	CS	PTA	MD	30,680	17,140/13540	All	①④⑤
Lin et al. (16)	2016	Taiwan	CS	HIS	MD	55,094	-	-	①③④⑤
Arslan et al. (18)	2018	Turkish	C-C	PTA	ADS (BAS)/DDS(BDI)	101	-	32.8 ± 9.9	④⑥
Crowson et al. (19)	2021	United States	C-S	PTA	SR (PHQ-9)	4,582	-	44.2 ± 14.3	④
Huang et al. (20)	2022	China	C-S	SR	SR (Depression) DDS (GDS-15)	8,962	2312/6650	60.2 ± 7.8	①④⑤
Liu et al. (21)	2022	China	CS	SR	DDS	13,690	–	58.7 ± 9.4	①③④⑤
Marmamula et al. (22)	2021	Indian	C-S	HIS	(SR) PHQ	867	330/537	74.2 ± 8.2	①④
Paiva et al. (23)	2023	Brazilian	C-S	SR	SR	1,335	510/825	≥60	④
Golub et al. (24)	2020	United States	CS	PTA	DDS (CESD-10)	5,499	2115/3384	58.6 ± 6.3	①
Lawrence et al. (25)	2019	Australia	C-S	PTA	MD	147,148	60,787/86361	73.43	①
Guan et al. (26)	2022	China	C-C	SR	DDS (CES-D-10)	14,455	7034/7421	61.93 ± 7.76	①

-: unclear; C-C: Case–control; C-S: Cross-sectional; CS: Cohort study; PTA: pure tone audiometry; HIS: hearing impairment scale; SR:self-report; DDS: depression diagnostic scale; MD: medical diagnosis; ADS: anxiety diagnostic scale; ① risk of depression among SNL patients ② risk of anxiety among SNL patients; ③ risk of SNL in patients with depression; ④ prevalence of depression among SNL patients; ⑤ prevalence of SNL in patients with depression; ⑥ prevalence of anxiety among SNL patients; ⑦ prevalence of SNL in patients with anxiety disorders.

### 3.2 Quality assessment and publication bias

Cross-sectional studies were evaluated using AHRQ criteria and included studies which explicitly stated the research question, specified the target study population and utilized valid and reliably administered exposure and outcomes measures. The results of the quality assessment indicated that no high-risk studies were included through detailed review, which are shown in Table 2.

**Table 2:** Quality assessment of included studies (Cohort and Case–control studies).

First author	Year	Selection	Comparability	Outcome	Overall quality score
Cohort studies					
Hsu et al. (8)	2016	★★	★★	★★★	7
Kim et al. (10)	2020	★★	★★	★★★	7
Powell et al. (14)	2021	★★	★★	★★	6
Kim et al. (15)	2017	★★	★★	★★	6
Lin et al. (16)	2016	★★★	★★	★★★	8
Liu et al. (21)	2022	★★	★★★	★★	7
Golub et al. (24)	2020	★★	★★	★★	6
Case control studies					
Tseng et al. (6)	2016	★★	★★	★★	6
Kim et al. (7)	2018	★★	★★★	★★★	8
Wu et al. (9)	2021	★★	★★	★★★	7
Chung et al. (11)	2015	★★	★★	★★	6
Arslan et al. (18)	2018	★★	★★	★★	6
Guan et al. (26)	2022	★★	★★	★★	6

The Newcastle-Ottawa Quality Assessment Scale (NOS) (27) was used to assess the quality of the included studies in three aspects, selection, comparison and results. The scores of cohort studies and case–control studies ranged from 0 to 9 and the higher the score, the higher the research quality. NOS scores ≥ 7, 4–6 and 0–3 represent high, medium and low quality, respectively.

Assessment of all the included cohort and case–control studies using the NOS scores indicated that 6 studies were of high quality, 7 studies were of moderate quality and no low-quality studies were included in this meta-analysis (Table 3).

**Table 3:** Quality assessment of included studies (Cross-sectional studies).

First Author-Year	Cejas et al. (12) 2021	Yang et al. (13) 2021	Crowson et al. (19) 2021	Huang et al. (20) 2022	Srinivas et al. (22) 2021	Paiva et al. (23) 2023	Lawrence et al. (25) 2019
I	Y	Y	Y	Y	Y	Y	Y
II	Y	Y	Y	Y	Y	Y	Y
III	U	Y	Y	Y	N	Y	N
IV	U	N	U	N	U	Y	N
V	N	N	N	N	N	N	N
VI	Y	Y	Y	Y	Y	Y	N
VII	U	N	U	N	N	N	N
VII	N	N	N	U	Y	Y	N
IX	N	N	N	N	N	N	N
X	N	Y	U	Y	Y	Y	Y
XI	N	Y	N	Y	Y	Y	Y

Y: Yes; N: No; U: Unclear; I: Define the source of information (survey, record review); II: List inclusion and exclusion criteria for exposed and unexposed subjects (cases and controls) or refer to previous publications; III: Indicate time period used for identifying patients; IV: Indicate whether or not subjects were consecutive if not population-based; V: Indicate if evaluators of subjective components of study were masked to other aspects of the status of the participants; VI: Describe any assessments undertaken for quality assurance purposes (e.g., test/retest of primary outcome measurements); VII: Explain any patient exclusions from analysis; VII: Describe how confounding was assessed and/or controlled; IX: If applicable, explain how missing data were handled in the analysis; X: Summarize patient response rates and completeness of data collection; XI: Clarify what follow-up, if any, was expected and the percentage of patients for which incomplete data or follow-up was obtained.

The outcome variables were plotted in funnel plots for the number of included literatures ≥5, and combined with Egger’s and Begg’s tests (Table 4), which showed no publication bias was observed for our outcomes (Supplementary Figures S1–S4).

**Table 4:** Results of meta-analysis of the bidirectional relationship between sensorineural hearing loss and depression-anxiety disorder.

Endpoint	Included studies	Heterogeneity test		Effect model	Meta-analysis results		Publication bias	
		I^2^-value	*p*-value		ES (95%CI)	*P*-value	Egger’s	Begg’s
(a) SNL-DS	13 (6–10, 15, 16, 20–26)	69.1%	0.000	R	1.43 (1.32–1.55)	<0.001	0.084	0.161
(b) SNL-DS	14 (6–8, 12–16, 18–23)	99.86%	<0.001	R	0.17 (0.09–0.28)	<0.001	0.054	0.511
(a) DS-SNL	5 (7, 9, 10, 16, 21)	68.2%	0.014	R	1.30 (1.11,1.48)	<0.001	0.452	0.806
(b) DS-SNL	7 (6–8, 15, 16, 20, 21)	99.61%	<0.001	R	0.46 (0.28–0.65)	<0.001	0.951	1.000
(a) SNL-AD	3 (7, 8, 11)	86.1%	0.001	R	1.83 (1.42,2.24)	<0.001	-	-
(b) SNL-AD	4 (7, 11, 12, 18)	98.82%	<0.001	R	0.40 (0.24–0.57)	<0.001	-	-
AD-SNL	2 (7, 11)	0%	<0.001	F	0.31 (0.29–0.33)	<0.001	-	-

-: unnecessary; ES: effect size; (a) SNL-DS: risk of depression among SNL patients; (b) SNL-DS: prevalence of depression among SNL patients; (a) DS-SNL: risk of SNL in patients with depression; (b) DS-SNL: prevalence of SNL in patients with depression; (a) SNL-AD: risk of anxiety among SNL patients; (b) SNL-AD: prevalence of anxiety among SNL patients; AD-SNL: prevalence of SNL in patients with anxiety disorders. R: Random; F: Fixed.

### 3.3 Prevalence and risk of depression in SNL patients

**Figure 2:**
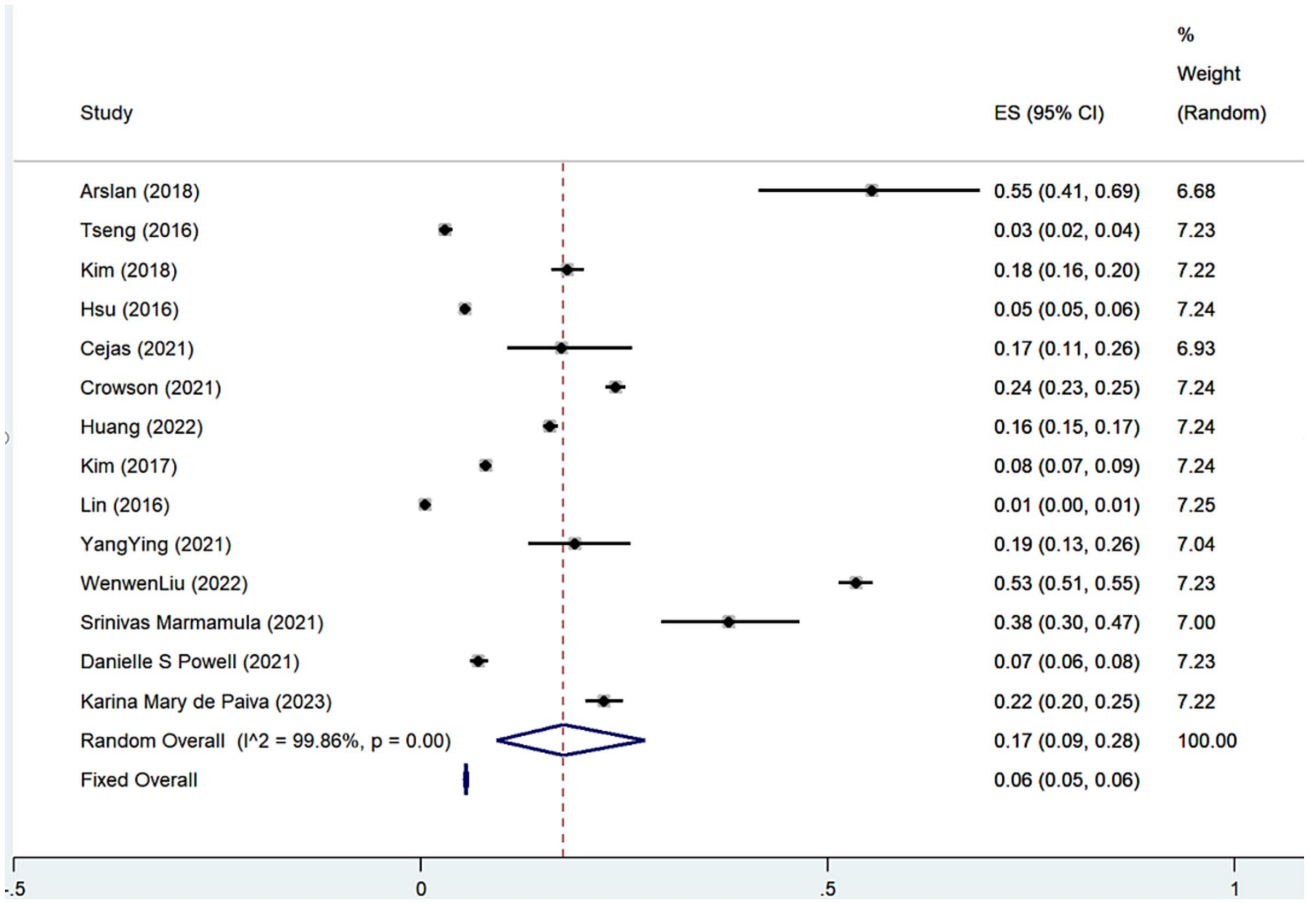
Forest plot of depression prevalence among patients with sensorineural hearing loss.

**Figure 3:**
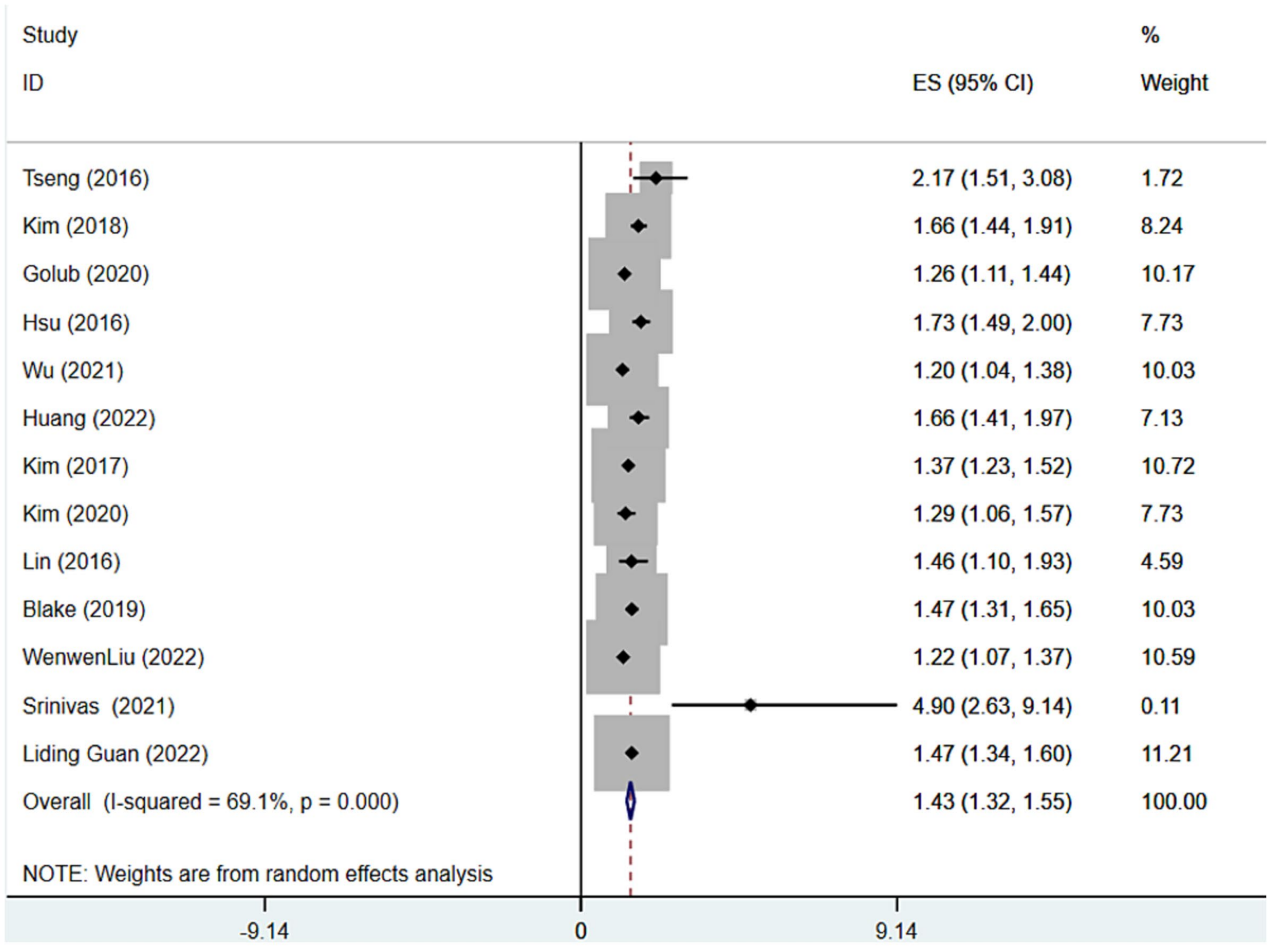
Forest plot of risk of depression among patients with sensorineural hearing loss.

A total of 14 studies (6–8, 12–16, 18–23) were presented on the prevalence of depression among SHL patients, and the results of the meta-analysis showed (Figure 2; Table 4) that the overall prevalence of SHL patients suffering from comorbid depression was 17% (Rate = 0.17, 95% CI: 0.09–0.28; *I*^2^ = 99.86%, *p* < 0.001), furthermore the meta-analysis of the 13 studies (6–10, 15, 16, 20–22, 24–26) indicated that SNL patients had higher risk of incidence of depression (Figure 3; Table 4) compared to the general population (OR = 1.43, 95% CI: 1.32–1.55; *I*^2^ = 69.1%, *p* = 0.000). Considering the relatively high heterogeneity of both findings, we conducted the sensitivity analysis (Supplementary Figures S5–S6) to explore whether the heterogeneity was sourced by excluding tests one-by-one, which showed that excluding any one piece of literature has no significant influence on the finding.

### 3.4 Prevalence and risk of SNL in depression patients

**Figure 4:**
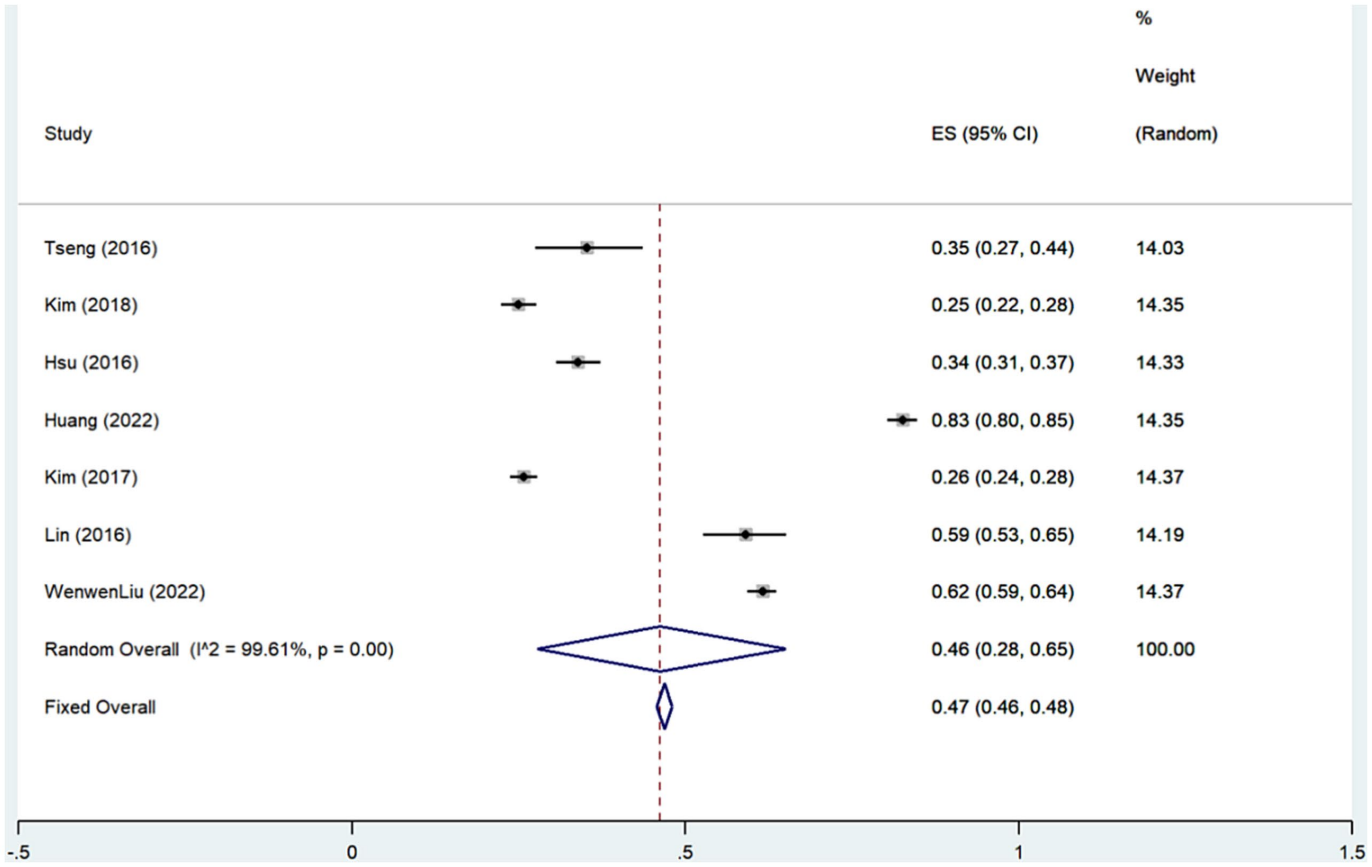
Forest plot of prevalence of sensorineural hearing loss in depressed patients.

**Figure 5:**
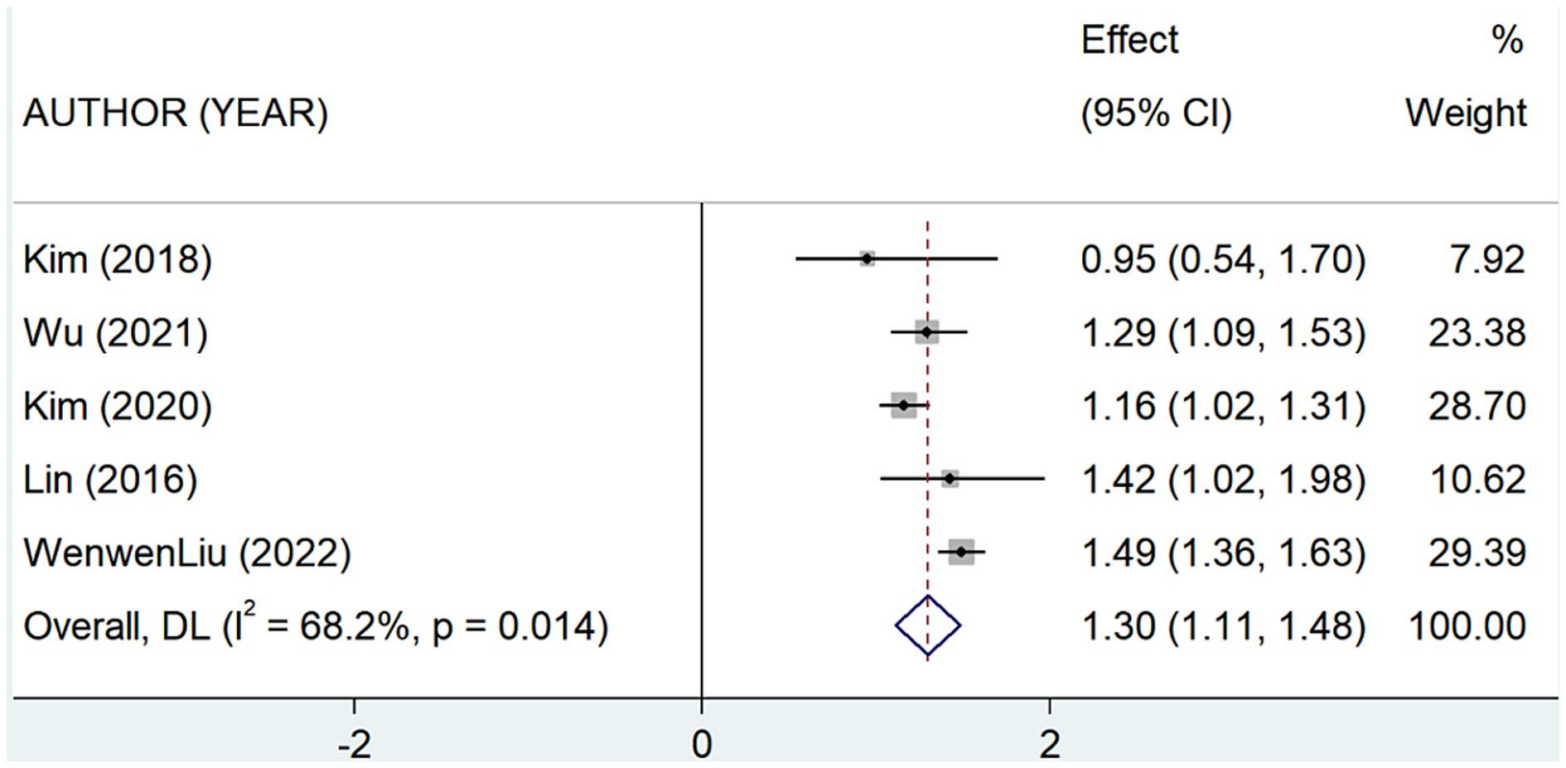
Forest plot of the risk of developing sensorineural hearing loss in depressed patients.

The current study included 7 articles [6-,8,15-16,20–21] assessing the prevalence of SNL in depressed patients, which suggest that the overall prevalence of SNL occurring in depressed patients was 46% (Rate = 0.46, 95% CI: 0.28–0.65; *I*^2^ = 99.61%, *p* < 0.001; Figure 4; Table 4). This heterogeneity could not be explained through sample size (large or small) or study design (prospective or retrospective), and sensitivity analyzes showed that none of the studies contributed significantly to the summary findings (Supplementary Figure S7). Combined 5-study (7, 9, 10, 16, 21) findings revealed that the risk of SNL prevalence is increased in patients with a history of depression (OR = 1.30, 95% CI: 1.11–1.48; *I*^2^ = 68.2%, *p* < 0.001; Figure 5; Table 4). The results of sensitivity analysis showed (Supplementary Figure S8) that the conclusion of Liu et al. (21) significantly deviated from the midline, and the heterogeneity was significantly reduced by excluding the findings of that study, and the recombined results using a fixed-effects model were (OR = 1.20, 95% CI: 1.09–1.32; *I*^2^ = 0%, *p* = 0.476).

### 3.5 Bidirectional association between SNL and anxiety disorders

**Figure 6:**
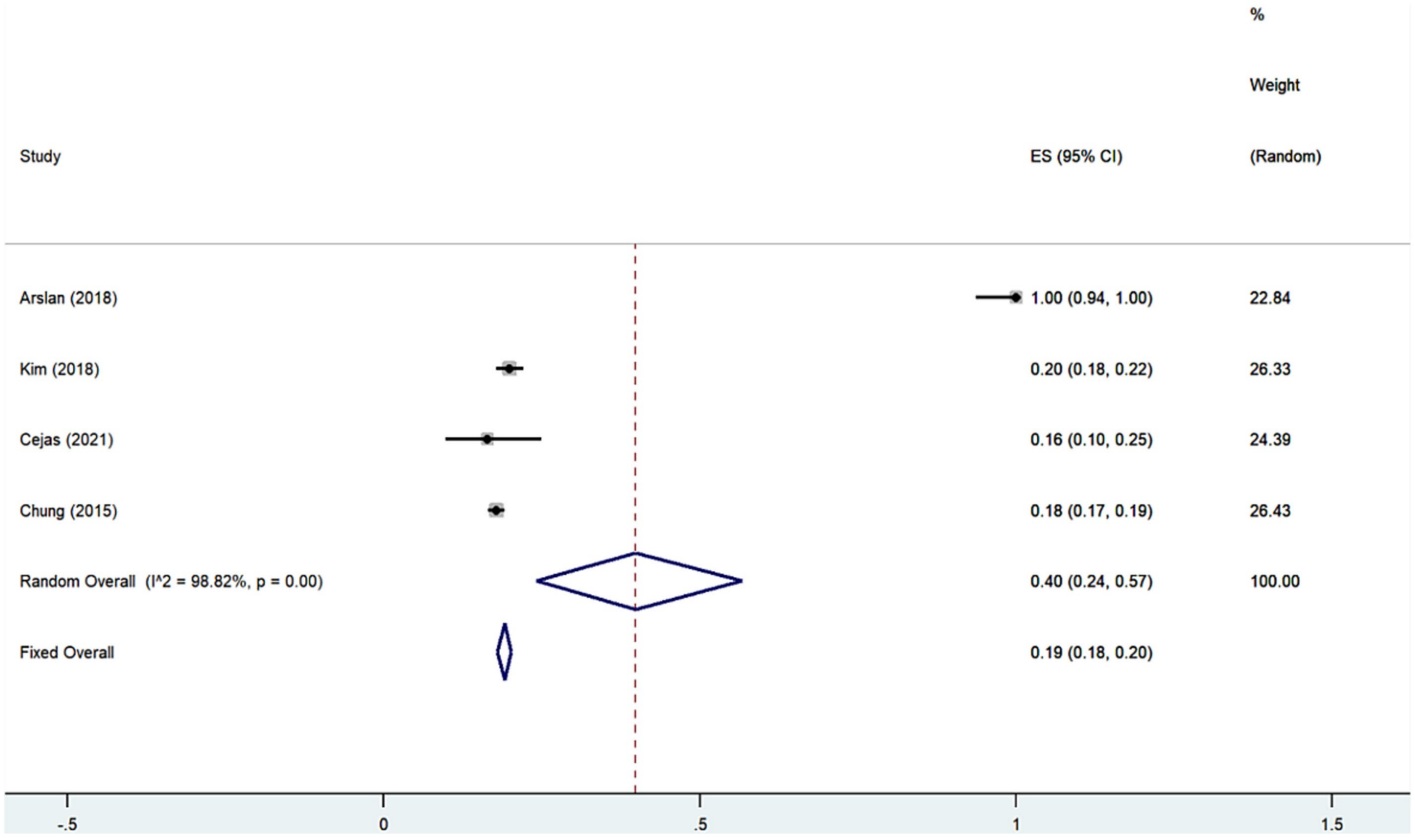
Forest plot of prevalence of anxiety disorders among patients with sensorineural hearing loss.

**Figure 7:**
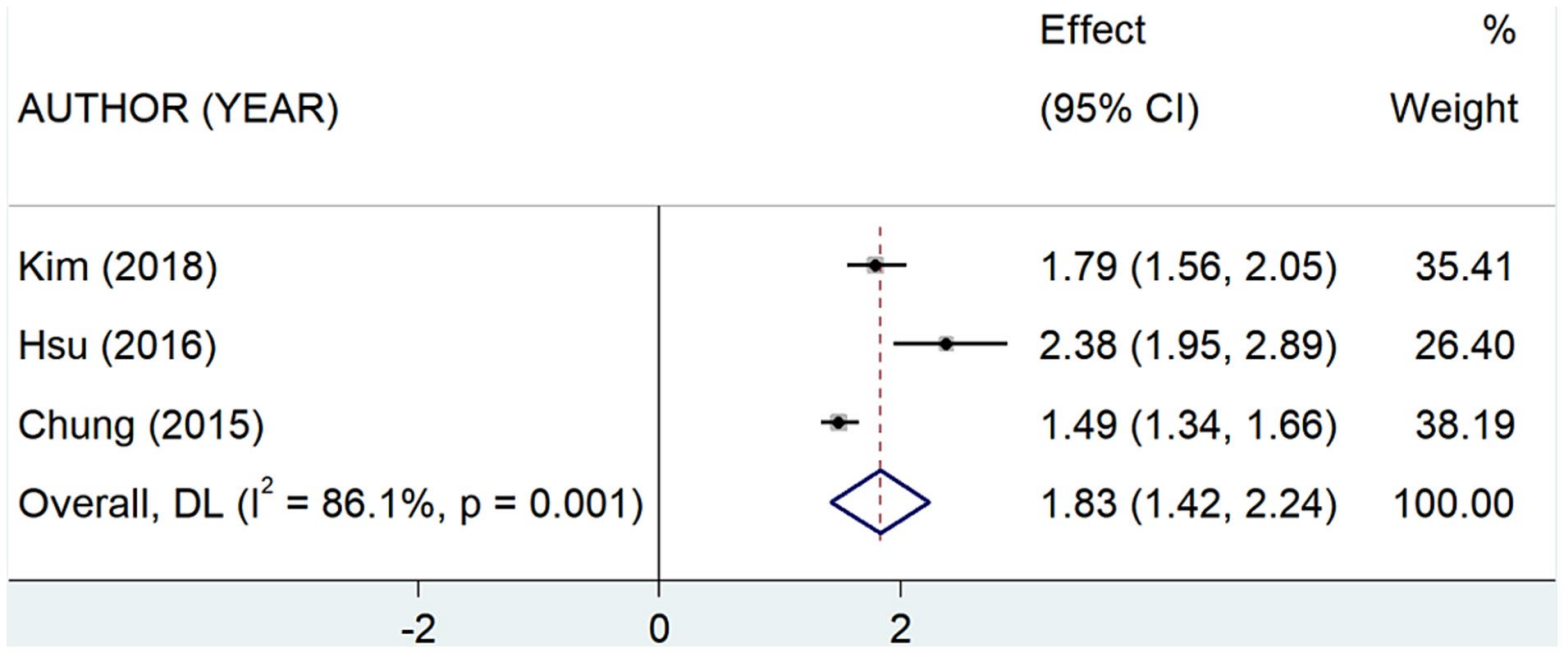
Forest plot of the risk of anxiety disorders in patients with sensorineural hearing loss.

We performed meta-analysis of the findings of the 4 studies (7, 11, 12, 18) indicating that the prevalence of anxiety disorders among SNL patients is approximately 40% (Rate = 0.40, 95% CI: 0.24–0.57; *I*^2^ = 98.82%, *p* < 0.001; Figure 6; Table 4), which is considerably high compared to the general population. Meanwhile, analyzing the results of study (7, 8, 11) showed that the risk of anxiety disorders in SNL patients is 1.83 times (OR = 1.83, 95% CI: 1.42–2.24; *I*^2^ = 86.1%, *p* < 0.001; Figure 7; Table 4) higher than that of the general population. Additionally, the prevalence of SNL in patients with anxiety disorders was 31% (OR = 0.31, 95% CI: 0.29–0.33; *I*^2^ = 0%, *p* < 0.001; Supplementary Figure S9; Table 4) (7, 11), suggesting perhaps the bidirectional association between the two disorders and the sensitivity analysis failed to find that none of the any one of included studies had influence on the conclusions (Supplementary Figures S10–S12).

### 3.6 Subgroup analysis

#### 3.6.1 Age

Fourteen studies discussed the percentage of SNL patients who developed depression and the results showed strong heterogeneity (*I*^2^ = 99.92%, *p* < 0.001), subgroup analysis was conducted according to age in the youth group (6–8, 12, 13, 15, 16, 18, 19) versus the older adults group (14, 20–23). The results of the combined Meta-analysis using the random-effects model showed that in the older adults group (Rate = 0.26, 95% CI: 0.11–0.44; *I*^2^ = 99.75%, *p* < 0.001) The percentage of SNL patients who developed depression was higher than that of the young group (Rate = 0.13, 95% CI (0.06–0.23); *I*^2^ = 99.81%, *p* < 0.001). Furthermore, it was essentially equal between the risk of depression in SNL patients in the younger (6–8, 10, 15, 16) (OR = 1.53, 95% CI: 1.35–1.71; *I*^2^ = 62.3%, *p* = 0.021) and older adults groups (20, 21, 24–26) (OR = 1.40, 95%CI: 1.26–1.53; *I*^2^ = 69.5%, *p* = 0.011), which was shown in Table 5. Seven studies discussed the percentage of depressed patients with hearing loss, which were categorized into young (6–8, 15, 16) and older adults (20, 21) groups according to age to carry out subgroup analyzes, with a significant decrease in heterogeneity in the old group (*I*^2^ = 0%, *p* < 0.001) and no significant change in the young group (*I*^2^ = 96.79%, p < 0.001). The results of the subgroup analyzes showed that a higher prevalence of SNL in the older adults group (OR = 0.70, 95% CI: 0.68–0.71; Table 5) depressed patients than in the youth group (OR = 0.35, 95% CI: 0.26–0.44; Table 5).

**Table 5:** Results of subgroup analysis (Age).

Endpoint (Age)	Included studies	Heterogeneity test		Effect model	Meta-analysis results	
		*I*^2^-value	*P*-value		ES (95%CI)	*P*-value
Older adults group						
(a) SNL-DS	5 (14, 20–23)	99.75%	<0.001	R	0.26 (0.11–0.44)	<0.001
(b)SNL-DS	5 (20, 21, 24–26)	69.5%	0.011	R	1.40 (1.26–1.53)	<0.001
(a) DS-SNL	2 (20, 21)	0%	<0.001	F	0.70 (0.68–0.71)	<0.001
Youth group						
(a) SNL-DS	9 (6–8, 12, 13, 15, 16, 18, 19)	99.81%	<0.001	R	0.13 (0.06–0.23)	<0.001
(b) SNL-DS	6 (6–8, 10, 15, 16)	62.3%	0.021	R	1.53 (1.35–1.71)	<0.001
(a) DS-SNL	5 (6–8, 15, 16)	96.79%	<0.001	R	0.35 (0.26–0.44)	<0.001

(a) SNL-DS: prevalence of depression among SNL patients; (b) SNL-DS: risk of depression among ES: effect size; SNL patients; (a) DS-SNL: prevalence of SNL in patients with depression. R: Random; F: Fixed.

#### 3.6.2 Region

We conducted subgroup analyzes based on geography which showed approximately equal percentages of depression in SNL patients between the Asian (6–8, 13, 15, 16, 20–22) and American (12, 14, 19, 23) groups (Table 6). Interestingly, the included European study (18) (Rate = 0.55) showed higher prevalence of depression in SNL patients compared to Asia (Rate = 0.15, 95% CI: 0.06–0.27; *I*^2^ = 99.89%, *p* < 0.001) and the Americas (Rate = 0.17; 95% CI: 0.08–0.28; *I*^2^ = 99.13%, *p* < 0.001), the difference that may be due to sample size. Furthermore, the risk of depression in the Asian group (7–10, 15, 16, 20, 21, 26) (OR = 1.43, 95%CI: 1.31–1.56; *I*^2^ = 70.7%, *p* = 0.001) of SNL patients was considerable compared with that of the Oceania group (25) (OR = 1.47, 95% CI: 1.31–1.65) and slightly higher than in the study of the Americas group (24) (OR = 1.26, 95% CI: 1.11–1.44).

**Table 6:** Results of subgroup analysis (Region).

Endpoint (Region)	Included studies	Heterogeneity test		Effect model	Meta-analysis results	
		*I*^2^-value	*P*-value		ES (95%CI)	*P*-value
Asian groups						
(a) SNL-DS	9 (6–8, 13, 15, 16, 20–22)	99.89%	<0.001	R	0.15 (0.06–0.27)	<0.001
(b) SNL-DS	9 (7–10, 15, 16, 20, 21, 26)	70.7%	0.001	R	1.43 (1.31–1.56)	<0.001
American groups						
(a)SNL-DS	4 (12, 14, 19, 23)	99.13%	<0.001	R	0.17 (0.08–0.28)	<0.001
(b) SNL-DS	1 (24)	-	-	-	1.26 (1.11–1.44)	-
European groups						
(a) SNL-DS	1 (18)	-	-	-	0.55	-
Oceania groups						
(b) SNL-DS	1 (25)	-	-	-	1.47 (1.31–1.65)	-

-: unnecessary; ES: effect size; (a) SNL-DS: prevalence of depression among SNL patients; (b) SNL-DS: risk of depression among SNL patients. R: Random; F: Fixed.

#### 3.6.3 Diagnostic modalities of SNL

Generally, the SNL diagnostic modalities of the studies we included were categorized into PTA group, Hearing Impairment Scale (HIS) group, and self-report (SR) group (Table 7). The results of subgroup analysis showed that the incidence of depression was highest in the SR group (13, 20, 21, 23) (Rate = 0.27, 95% CI: 0.10–0.48; *I*^2^ = 99.73%, *p* < 0.001) followed by the PTA group (6, 7, 12, 14, 15, 18, 19) (Rate = 0.16, 95% CI: 0.09–0.24; *I*^2^ = 99.36%, *p* < 0.001) and the HIS group (8, 16, 22) (Rate = 0.09, 95% CI: 0.02–0.20; *I*^2^ = 99.68%, *p* < 0.001) among SNL patients. Interestingly, patients diagnosed with SNL by HIS (8, 16) (OR = 1.66, 95% CI: 1.44–1.87; *I*^2^ = 15.10%, *p* = 0.278) had a slightly higher OR for depression than the PTA (6, 10, 15, 24, 25) (OR = 1.37, 95% CI: 1.28–1.45; *I*^2^ = 45.9%, *p* = 0.116) and SR (9, 20, 21, 26) (OR = 1.37, 95% CI: 1.18–1.55; *I*^2^ = 78.30%, *p* = 0.003). Additionally, the risk of developing SNL in depressed patients may be associated with the diagnostic modality of SNL, and the results of subgroup analyzes showed that the HIS group (16) (OR = 1.42, 95% CI: 1.02–1.98) and the SR (9, 21) (OR = 1.41, 95% CI: 1.22–1.60; *I*^2^ = 56.60%, *p* = 0.129) group had a roughly similar and higher SNL risk was broadly similar and higher than in the PTA group (7, 10) (OR = 1.15, 95% CI: 1.01–1.29; *I*^2^ = 0.00%, *p* = 0.491). The incidence of SNL among depressed patients also demonstrated differences among the subgroups, and the results showed that the SR (20, 21) group (Rate = 0.70, 95% CI: 0.68–0.71; *I*^2^ = 0.00%, *p* = 0.926) exhibited the highest prevalence of SNL followed by the HIS (8, 16) (Rate = 0.40, 95% CI: 0.37–0.43; *I*^2^ = 0.00%, *p* = 0.999) and PTA (6, 7, 15) (Rate = 0.27, 95% CI: 0.23–0.30; *I*^2^ = 69.57%, *p* = 0.04) groups. Risk of anxiety in patients with SNL disorders was lower in the PTA group (7, 11) (OR = 1.63, 95% CI: 1.33–1.92; *I*^2^ = 75.20%, *p* = 0.44) than in the HIS group (8) (OR = 2.38, 95% CI: 1.95–2.89).

**Table 7:** Results of subgroup analysis (Diagnostic modalities of SNL).

Endpoint	Included studies	Heterogeneity test		Effect model	Meta-analysis results	
		*I*^2^-value	*P*-value		ES (95%CI)	*P*-value
PTA group						
(a) SNL-DS	(6, 7, 12, 14, 15, 18, 19)	99.36%	<0.001	R	0.16 (0.09–0.24)	<0.001
(b) SNL-DS	5 (6, 10, 15, 24, 25)	45.9%	0.116	F	1.37 (1.28–1.45)	<0.001
(a) DS-SNL	3 (6, 7, 15)	69.57%	0.040	R	0.27 (0.23–0.30)	<0.001
(b) DS-SNL	2 (7, 10)	0.0%	0.491	F	1.15 (1.01–1.29)	<0.001
(b) SNL-AD	2 (7, 11)	75.2%	0.044	R	1.63 (1.33–1.92)	<0.001
HIS group						
(a) SNL-DS	3 (8, 16, 22)	99.68%	<0.001	R	0.09 (0.02–0.20)	<0.001
(b) SNL-DS	2 (8, 16)	15.10%	0.278	F	1.66 (1.44–1.87)	<0.001
(a) DS-SNL	2 (8, 16)	0.00%	0.999	F	0.40 (0.37–0.43)	<0.001
(b) DS-SNL	1 (16)	-	-	-	1.42 (1.02–1.98)	-
(b) SNL-AD	1 (8)	-	-	-	2.38 (1.95–2.89)	-
SR group						
(a) SNL-DS	4 (13, 20, 21, 23)	99.73%	<0.001	R	0.27 (0.10–0.48)	<0.001
(b) SNL-DS	4 (9, 20, 21, 26)	78.30%	0.003	R	1.37 (1.18–1.55)	<0.001
(a) DS-SNL	2 (20, 21)	0.00%	0.926	F	0.70 (0.68–0.71)	<0.001
(b) DS-SNL	2 (9, 21)	56.6%	0.129	R	1.41 (1.22–1.60)	<0.001

-: unnecessary; ES: effect size; PTA: pure tone audiometry; HIS: hearing impairment scale; SR: self-report; (a) SNL-DS: prevalence of depression among SNL patients; (b) SNL-DS: risk of depression among SNL patients; (a) DS-SNL: prevalence of SNL in patients with depression; (b) DS-SNL: risk of SNL in patients with depression (b) SNL-AD: risk of anxiety among SNL patients. R: Random; F: Fixed.

#### 3.6.4 Diagnostic modalities of depression and anxiety

Subgroup analysis (Table 8) was conducted by dividing the study into medical diagnosis (MD) group, depression scale (DS) group, and self-report (SR) group based on the mode of depression diagnosis, and the results showed that the risk of developing depression was higher in the SR (22) (OR = 4.90, 95% CI: 2.63–9.14) group than the risk of developing depression in the MD (6, 7, 10, 15, 16, 25) (OR = 1.45, 95% CI: 1.36–1.54; *I*^2^ = 44.6%, *p* = 0.108) group and DS (9, 20, 21, 24) (OR = 1.30, 95% CI: 1.15–1.46; *I*^2^ = 64.9%, *p* = 0.036) group among SNL patients. Nevertheless, the incidence rate perspective analysis of the outcome showed that the percentage of SNL occurrence was lower in the MD (6, 7, 15, 16) (OR = 0.06, 95% CI: 0.01–0.15; *I*^2^ = 99.80%, *p* < 0.001) group than in the DS (8, 13, 14, 18, 20, 21) (Rate = 0.23, 95% CI: 0.09–0.40; *I*^2^ = 99.79%, *p* = 0.00) group and in the SR (12, 19, 22, 23) (Rate = 0.24, 95% CI: 021–0.28; *I*^2^ = 81.79%; *p* = 0.00) groups. The results of the subgroup analysis confirmed that the OR of having SNL among depressed patients was slightly higher in the MD (7, 10, 16) group (OR = 1.17, 95% CI: 1.03–1.30; *I*^2^ = 0.0%, *p* = 0.436) than in the DS (9, 21) group (OR = 1.41, 95% CI: 1.22–1.60; *I*^2^ = 56.6%, *p* = 0.129). The prevalence of SNL among depressed patients was different depending on the diagnostic modality of depression, with a high prevalence in the DS group (8, 20, 21) (Rate = 0.60, 95% CI: 0.35–0.83; *I*^2^ = 99.61%, *p* = 0.00), followed by the MD group (6, 7, 15) (OR = 0.27, 95% CI: 0.23–0.30%; *I*^2^ = 69.57%, *p* = 0.04). Moreover, the diagnostic modality of anxiety disorders in SNL patients also influenced the OR of patients developing anxiety disorders, with the diagnostic group relying on the Anxiety Scale (8) (OR = 2.38, 95% CI: 1.95–2.89) relative to the MD group (7, 11) (OR = 1.63, 95% CI: 1.33–1.92; *I*^2^ = 75.2%, *p* = 0.044) the OR was higher. Regarding the prevalence of anxiety disorders in SNL patients, relationships between the Anxiety Scale diagnostic group (12, 18) (Rate = 0.52, 95% CI: 0.44–0.59; *I*^2^ = 0.0%, *p* = 0.997) and the MD (7, 11) (Rate = 0.18, 95% CI: 0.17–0.19; *I*^2^ = 0.0%, *p* = 0.996) group relations showed the similar trend.

**Table 8:** Results of subgroup analysis (Diagnostic modalities of depression and anxiety).

Endpoint	Included studies	Heterogeneity test		Effect model	Meta-analysis results	
		*I*^2^-value	*P*-value		ES (95%CI)	*P*-value
MD group						
(a) SNL-DS	4 (6, 7, 15, 16)	99.80%	< 0.001	R	0.06 (0.01–0.15)	<0.001
(b) SNL-DS	6 (6, 7, 10, 15, 16, 25)	44.6%	0.108	F	1.45 (1.36–1.54)	<0.001
(a) DS-SNL	3 (6, 7, 15)	69.57%	0.04	R	0.27 (0.23–0.30)	<0.001
(b) DS-SNL	3 (7, 10, 16)	0.0%	0.436	F	1.17 (1.03,1.30)	<0.001
(a) SNL-AD	2 (7, 11)	0.0%	0.996	F	0.18 (0.17–0.19)	<0.001
(b) SNL-AD	2 (7, 11)	75.2%	0.044	R	1.63 (1.33–1.92)	<0.001
DS/AD group						
(a) SNL-DS	6 (8, 13, 14, 18, 20, 21)	99.79%	0.00	R	0.23 (0.09–0.40)	<0.001
(b) SNL-DS	4 (9, 20, 21, 24)	64.9%	0.036	R	1.30 (1.15–1.46)	<0.001
(a) DS-SNL	3 (8, 20, 21)	99.61%	0.00	R	0.60 (0.35–0.83)	<0.001
(b) DS-SNL	2 (9, 21)	56.6%	0.129	R	1.41 (1.22,1.60)	<0.001
(a) SNL-AD	2 (12, 18)	0.0%	0.997	F	0.52 (0.44–0.59)	<0.001
(b) SNL-AD	1 (8)	-	-	-	2.38 (1.95–2.89)	-
SR group						
(a) SNL-DS	4 (12, 19, 22, 23)	81.79%	0.00	R	0.24 (021–0.28)	<0.0001
(b) SNL-DS	1 (22)	-	-	-	4.90 (2.63–9.14)	-

-: unnecessary; MD: medical diagnosis; DS: depression scale; SR: self-report; (a) SNL-DS: prevalence of depression among SNL patients; (b) SNL-DS: risk of depression among SNL patients; (a) DS-SNL: prevalence of SNL in patients with depression; (b) DS-SNL: risk of SNL in patients with depression; (a) SNL-AD: prevalence of anxiety among SNL patients; (b) SNL-AD: risk of anxiety among SNL patients. R: Random; F: Fixed.

## 4 Discussion

In recent years, the association between SNL and depression and anxiety disorders has emerged as a major focus of scholarly study. There is growing evidence of association between SNL and psychiatric disorders including depression, anxiety disorders, and cognitive impairment which begins earlier than previously recognized (subclinical stage of normal hearing) (28–30).

Nevertheless, some controversy may exist regarding the conclusions, and therefore the integration of the studies’ conclusions to provide reference for clinical diagnosis and treatment could be necessary. To the best of our knowledge, no meta-analysis systematically addressed the bi-directional association of SHL with depression and anxiety disorders in any of the studies up to now, especially the risk and incidence of the development of comorbid SNL in patients with depression or anxiety disorders. Therefore, we performed a meta-analysis based on the bidirectional association of SHL with depression and anxiety disorders in expectation of providing further evidence-based medical evidence for clinical practitioners.

The results of our study showed an increased prevalence and risk of depression in subjects with SNL compared to normal hearing subjects. Several population-based studies have shown that people with SHL have higher risk of depression than those with normal hearing (6–8, 10), which is similar to the conclusions of our study. Furthermore, our analysis of the combined results of the included studies confirmed that the prevalence of SNL and the risk of developing SNL were also increased in depressed patients, suggesting that perhaps the bidirectional association exists between SNL and depression. Individuals with hearing loss may experience communication difficulties (31), social and emotional isolation (32), and affective disorders (7), all of which are independently associated with the development of depressive symptoms (7, 31, 32). A clinical trial demonstrated that depression symptoms are controlled in SNL patients who provide interventions to treat (26). Moreover, recent studies have shown that audiological rehabilitation, including the use of sound amplification devices (31), and the availability of hearing aids and sound amplification devices (33), which probably would have slowed down the incidence of depression in SNL patients. Besides, SHL patients commonly suffer from varying degrees of social isolation, which may increase the development of depressive symptoms (34–36). The existence of a link between social isolation/loneliness has been found to decrease regional brain volume perception in areas that support emotional processing and socialization (37). Similarly, patients with depression and anxiety disorders are usually under stress, and increased sensitivity or anxiety in stressed individuals may sensitize them to the perception of hearing loss (38). Liu et al. showed that the risk of SNL in depressed patients was 1.49 folds that in normal individuals, which is similar to our findings (21). On the other hand, social isolation has been associated with abnormalities in ventral striatal function, with worse connectivity in response to social information and dysfunctional frontal limbic emotion processing, which may be a contributing factor to the development of depressive symptoms in SNL patients (39).

Meanwhile, the risk and prevalence of anxiety disorders were found to be elevated in patients with SNL, and the prevalence of SNL was also identified to be increased in patients with anxiety disorders, which may demonstrate that there is a bilateral link between SNL and anxiety disorders. Kevin et al.’s findings suggest that the association between SNL and anxiety disorders is perhaps mediated via other mental health factors (e.g., social isolation and sensory deprivation) (40). Fatih et al. indicated that patients with SNL had significantly elevated anxiety scores relative to the general population, which is consistent with the findings of our study (18). Additionally, amygdala is a critical structure involved in several emotional functions and has been associated with multiple mood disorders, including anxiety and depression (41). The results of several studies suggest (42, 43) that SNL may cause abnormal neural responses in the amygdala. The amygdala’s decreased response to emotional stimulation in patients with SHL (43, 44) suggests that long-term hearing loss could diminish the transmission of auditory and emotional valence information by affecting the pattern of connectivity between the auditory cortex and the amygdala. Tang et al. (45) showed that SHL impairs temporal synchronization between the amygdala and the striatum. Such abnormalities ultimately can result in abnormal responses to emotionally significant stimuli or even emotional deficits in patients with SHL, which could lead to the development of anxiety disorders.

Age has been recognized as a marker of negative prognosis in a variety of disorders, and our findings suggest that older patients with depression or/and SNL experience greater risk and incidence of SNL or/and depression than the younger population. Hearing impairment is commonly recognized as a natural part of the aging process and the older adults tend to be more vulnerable to hearing loss than the younger population (46). Suzanne et al. showed that declines in social communication and activities of daily living in older patients with SNL were identified as a significant factor contributing to poorer mental health outcomes (47). Several studies have confirmed that the prevalence of depression in older adults SNL patients rises dramatically over time (48, 49). Similarly, depression-induced social communication deficits and social withdrawal may further amplify the role of social isolation in SNL development. The treatment of SNL may reverse or reduce symptoms of depression, especially in the older adults population. Mulrow et al. reported significant reductions in depressive symptoms in older adults patients at 6 weeks and 4 months after wearing hearing aids, while quality of life and cognitive functioning increased significantly. The treatment of SNL has been shown to be effective in the treatment of depression in the older adults, particularly in the older adults population (50, 51).

While our study suggests bidirectional relationship between SNL and depression. Interestingly, differences may exist between different geographic regions. Despite the fact that the majority of the studies we included were conducted based on Asian populations, nevertheless, subgroup analyzes showed that the incidence of depression was roughly the same among SNL patients in Asia and the Americas. Patients with SNL in Asia, however, experienced a higher risk of depression than in the Americas. This difference is perhaps due in part to differences in racial composition. A genome-wide association study based on depression suggests that depression-related genetic variant sites differ between individuals of East Asian and European ancestry (52). On the other hand, since the onset of depression is partially correlated with an individual’s social background, economic level, and level of medical care, the greater number of developing countries in Asia may partially explain the higher risk of depression in SNL patients (53). Further studies are necessary to continue to explore the influence of region on the bidirectional association between SNL and depression.

In our study, we discovered that SNL and diagnostic methods for depression and anxiety disorders perhaps influenced the results of the meta-analysis. As we described SNL diagnosed based on self-report and the Hearing Impairment Scale had a modest increased risk of depression onset relative to the PTA diagnostic group. Prevalence differences in depression between different SNL diagnostic groups could be attributed to the fact that we included studies of younger populations, who typically perceive their hearing impairment more accurately than older adults populations. A population-based cross-sectional study showed that the sensitivity of self-reported hearing loss in older adults was 41–65% (54). However, a prospective study that included younger subjects noted sensitivity of 81% for PTA as a diagnostic criterion for hearing loss (55). In addition, ethnicity, socioeconomic status, and educational attainment may perhaps have partially influenced the results, attributable to the fact that most of the studies we included were conducted in Asian countries. Higher levels of education have been associated with concordance between self-reported hearing loss and audiometric PTA results in several studies that have shown that age and education are associated with the correct perception of hearing impairment (47, 56, 57). The prevalence of self-reported hearing loss and audiometric hearing loss is higher in low-education and/or low-income populations (58). The greater accessibility of healthcare content and services in higher education groups may influence the concordance between self-reported hearing loss and audiometric hearing (58). The results of our subgroup analyzes showed that the prevalence of SNL was found to be higher in the population with self-reported diagnosis of depression and/or anxiety disorders compared to the scale-diagnosed group as compared to the medical-diagnosed group. Kim et al. (59) demonstrated that anxiety and depression showed significant correlation with overestimation of hearing loss. On the other hand, hearing impairment has adverse influence on depression and cognition, especially in the older adults (60, 61). Moreover, depression and anxiety have been reported to be strongly correlated with self-reported hearing loss (61, 62).

To the best of our knowledge, this meta-analysis constitutes the first attempt to argue for the bidirectional association between SNL and depression and anxiety disorders in the context of prevalence and risk of incidence. Our meta-analysis was based on comprehensive search that included 3 databases, and extensive manual searches were conducted for more comprehensive literature screening. Furthermore, we performed numerous analyzes and conducted subgroup studies seeking to demonstrate bi-directional associations between SNL and depression and anxiety disorders previously from multiple perspectives. Another strength of this study is the large sample size included, we had included altogether 675,291 patients including from 4 continents, which makes our results more credible and generalizable. The majority of the studies we included were characterized by high quality, most of which were adjusted for multiple confounders, possessed higher levels of evidence, and demonstrated efficacy in increasing the potential association of disease. However, several limitations should be recognized as well in our study, which considered with criticality. Although we included 20 studies for analysis, nevertheless the findings were relatively scattered, especially in targeting the association of anxiety disorders with SNL. Furthermore, despite the considerable amount of sensitivity analyzes and subgroup analyzes we performed, partially unexplained heterogeneity still existed, which may be attributed to differences in study design methods and selection of populations. The studies in the meta-analysis partly assessed depression and anxiety by using self-report questionnaires, however self-report questionnaires lacked interpretability and had lower specificity in identifying depression and anxiety. Meanwhile, older adults depressed individuals typically present more frequently with somatic symptoms, which may not be identified by general screening tools, and reliance on self-reported data on hearing ability may introduce bias. Secondly, some studies have utilized cross-sectional designs, which perhaps leads to an inability to pinpoint disease causation and accurately assess individuals when symptoms are present. Perhaps more importantly for the typing aspect of SNL, we attempted to analyze SNL in subgroups of age-related deafness and sudden deafness, regrettably some studies we included were unable to accurately characterize the type of SNL the patients suffered from. We continue to expect further studies to elaborate our findings from the perspective of SNL typing (age-related deafness and sudden deafness) in the future. Due to the large number of findings, the available evidence for subgroup analyzes had a relatively small number of subgroups and significant heterogeneity in the literature for each subgroup design. Further research is needed to understand the mechanisms underlying the relationship, especially research that demonstrates the link between dose–response relationships. Even so, regardless of these limitations, our analysis is extremely significant. This meta-analysis has revealed, for the first time, the bidirectional association between SNL and depressives and anxiety disorders in terms of prevalence and risk of onset, which can contribute to the identification of SNL and susceptible populations of depressives and anxiety, and provide new strategies for prevention and early intervention in the development of the disorders.

## 5 Conclusion

The current study found the bidirectional relationship between SNL and depression-anxiety disorders. Nevertheless, published reports are still relatively underdeveloped. Further studies are required to understand the mechanisms underlying the relationship and to conduct detailed subgroup analyzes for typing between disorders, especially to demonstrate the influence of dose–response on the relationship between disorders.

## Data availability statement

The original contributions presented in the study are included in the article/Supplementary material, further inquiries can be directed to the corresponding author.

## Author contributions

Z-qZ: Conceptualization, Data curation, Formal analysis, Investigation, Methodology, Project administration, Resources, Software, Supervision, Validation, Visualization, Writing – original draft, Writing – review & editing. J-yL: Conceptualization, Investigation, Methodology, Project administration, Supervision, Validation, Writing – review & editing. S-tG: Conceptualization, Investigation, Methodology, Methodology, Writing – review & editing. T-yM: Conceptualization, Formal analysis, Investigation, Project administration, Writing – review & editing. F-yL: Conceptualization, Conceptualization, Methodology, Writing – review & editing. J-lL: Conceptualization, Investigation, Methodology, Validation, Writing – review & editing. S-rS: Investigation, Methodology, Supervision, Validation, Writing – review & editing. Z-zC: Investigation, Methodology, Software, Writing – review & editing. Y-lJ: Data curation, Funding acquisition, Methodology, Supervision, Writing – review & editing. X-hJ: Conceptualization, Data curation, Investigation, Methodology, Project administration, Writing – review & editing.
