# Supplementary material for: Bidirectional associations between sensorineural hearing loss and depression and anxiety: a meta-analysis
Source: Front Public Health. 2024 Jan 8;11:1281689. doi: 10.3389/fpubh.2023.1281689 (PMC10800407; doi:10.3389/fpubh.2023.1281689)
Supplement: Supplementary file 1 [file Table_1.doc]

*Supplementary Material (Tables)*

Supplementary Table 1. Literature Retrieval Strategy.

| **PubMed** | | |
| --- | --- | --- |
| #1 | Search(((((Hypoacusis[Title/Abstract]) OR (Hearing Loss[Title/Abstract])) OR (Hypoacusis[Title/Abstract])) OR (Hearing Impairment[Title/Abstract])) OR (Deafness[Title/Abstract])) OR (Transitory Hearing Losses[Title/Abstract]) | 81,313 |
| #2 | Search (((Depression[Title/Abstract]) OR (Depressive Symptom[Title/Abstract])) OR (Emotional  Depression[Title/Abstract]))OR (Melancholia[Title/Abstract]) | 429,443 |
| #3 | Search((((Anxiety[Title/Abstract]) OR (Angst[Title/Abstract])) OR (Hypervigilance[Title/Abstract])) OR (Nervousness[Title/Abstract])) OR (Anxiousness[Title/Abstract]) | 266,941 |
| #4 | #2 OR #3 | 571,322 |
| #5 | #1 AND #4 | 1,515 |
| **Embase** | | |
| #1 | 'Hypoacusis':ab,ti OR 'Hearing Loss':ab,ti OR 'Hypoacuses':ab,ti OR 'Hearing Impairment':ab,ti OR 'Deafness':ab,ti OR 'Transitory Hearing Losses':ab,ti | 97,069 |
| #2 | 'Depression':ab,ti OR 'Depressive Symptom':ab,ti OR 'Emotional  Depression':ab,ti OR 'Melancholia':ab,ti | 577,480 |
| #3 | 'Anxiety':ab,ti OR 'Angst':ab,ti OR 'Hypervigilance':ab,ti OR 'Nervousness':ab,ti OR 'Anxiousness':ab,ti | 371,738 |
| #4 | #2 OR #3 | 770,224 |
| #5 | #1 AND #4 | 1,957 |
|  | **Web of Science** |  |
| #1 | Search TS=(Hypoacusis OR Hearing Loss OR Hypoacuses OR Hearing Impairment OR Deafness OR Transitory Hearing Losses) | 76,873 |
| #2 | Search TS=(Depression OR Depressive Symptom OR Emotional Depression OR Melancholia) | 629,982 |
| #3 | Search TS=(Anxiety OR Angst OR Hypervigilance OR Nervousness OR Anxiousness) | 376,798 |
| #4 | #2 OR #3 | 821,317 |
| #5 | #1 AND #4 | 2,349 |
