# Supplementary material for: Bidirectional associations between sensorineural hearing loss and depression and anxiety: a meta-analysis
Source: Front Public Health. 2024 Jan 8;11:1281689. doi: 10.3389/fpubh.2023.1281689 (PMC10800407; doi:10.3389/fpubh.2023.1281689)
Supplement: Supplementary file 2 [file Image_1.PDF]

Supplementary Material (Figures)

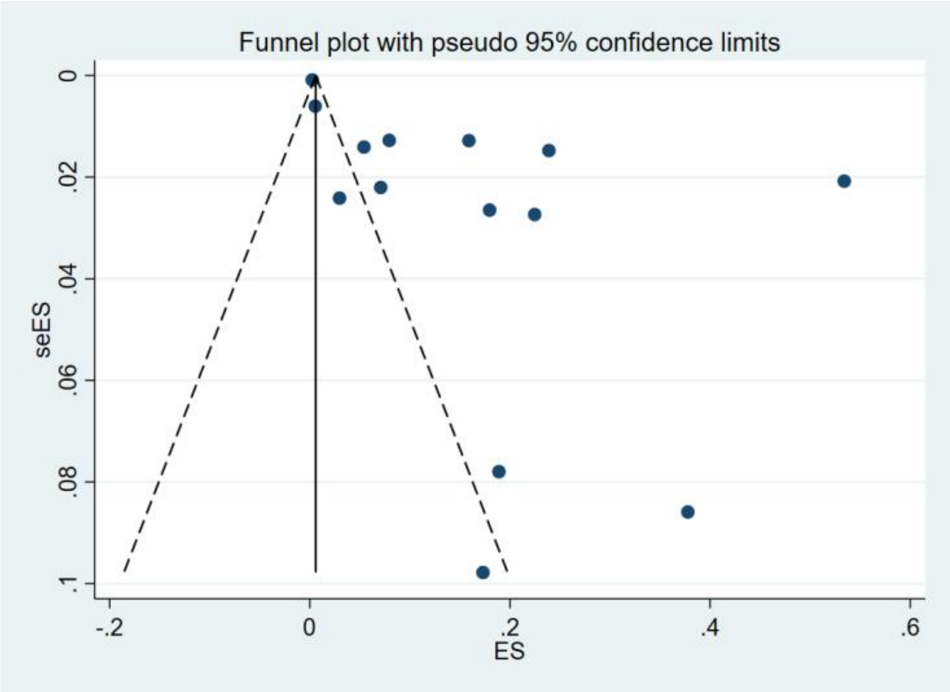

Supplementary Figure 1. Funnel plot of the prevalence of depression among patients with sensorineural hearing loss

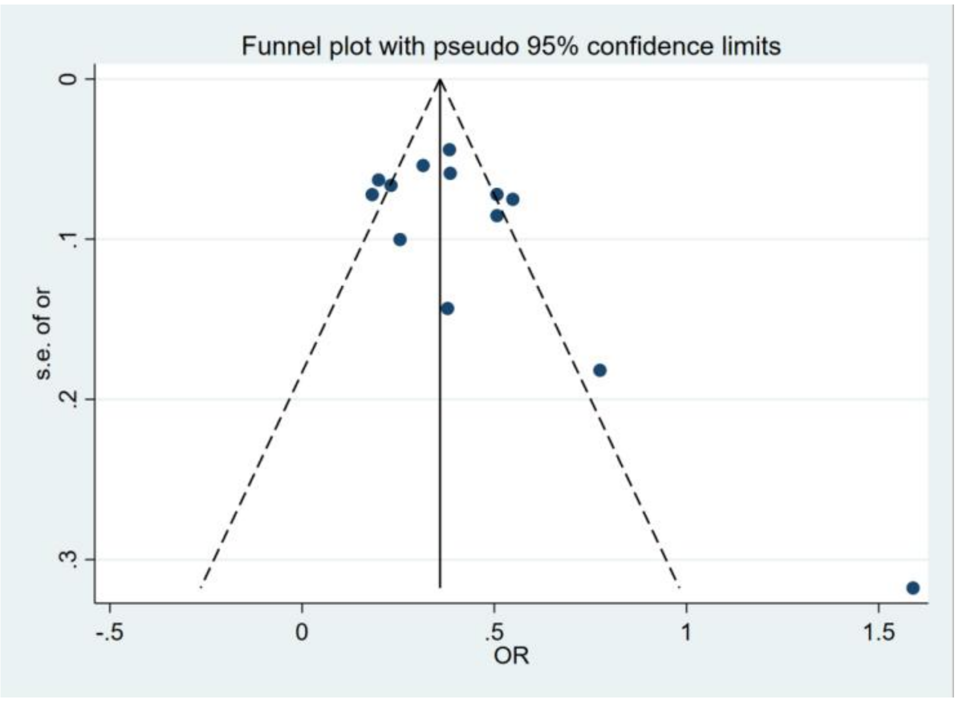

Supplementary Figure 2. Funnel plot of the risk of depression among patients with sensorineural hearing loss

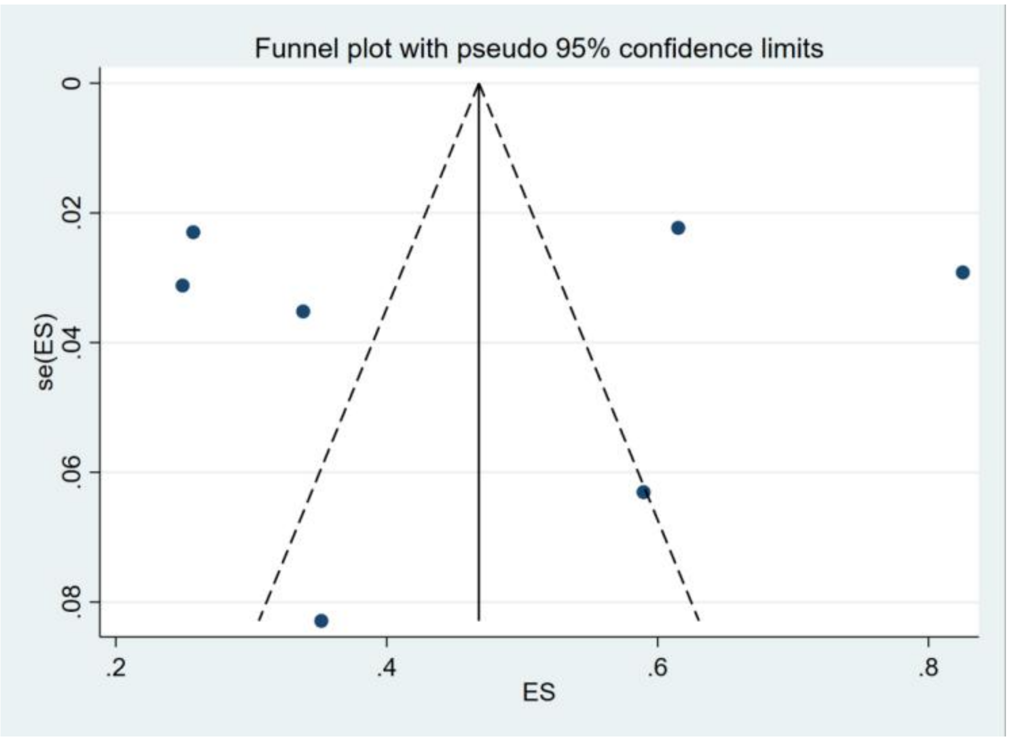

Supplementary Figure 3. Funnel plot of the prevalence of sensorineural hearing loss among depressed patients

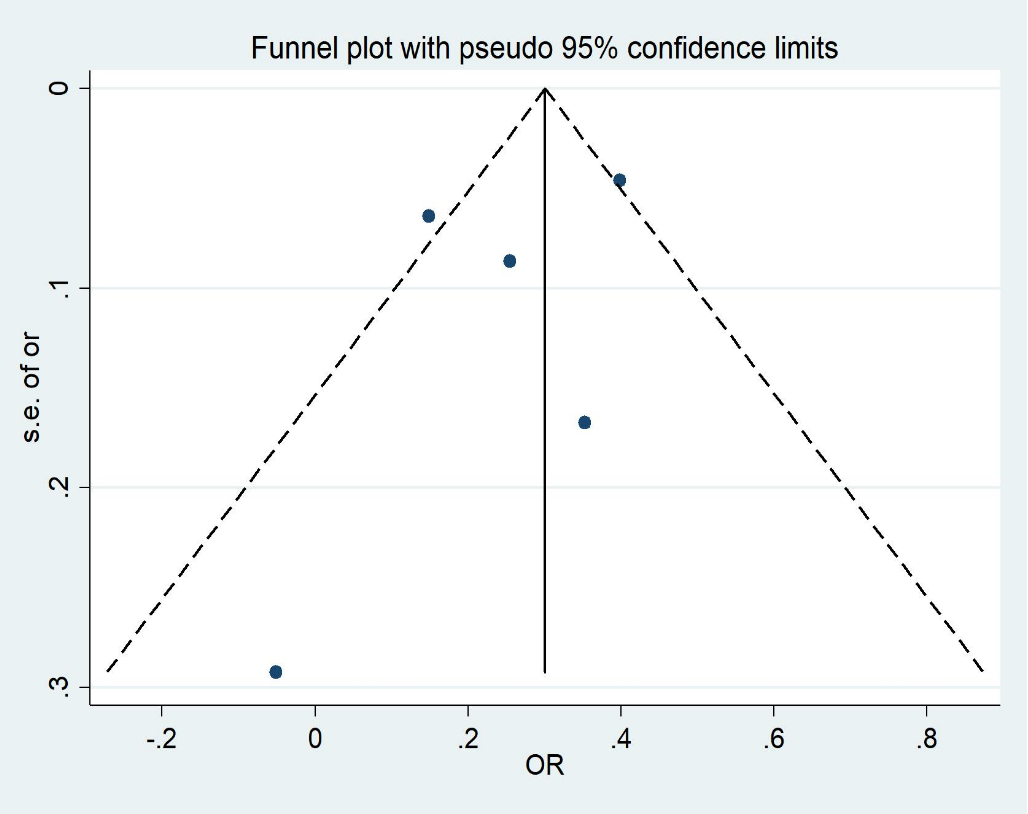

Supplementary Figure 4. Funnel plot of the prevalence of sensorineural hearing loss among depressed patients

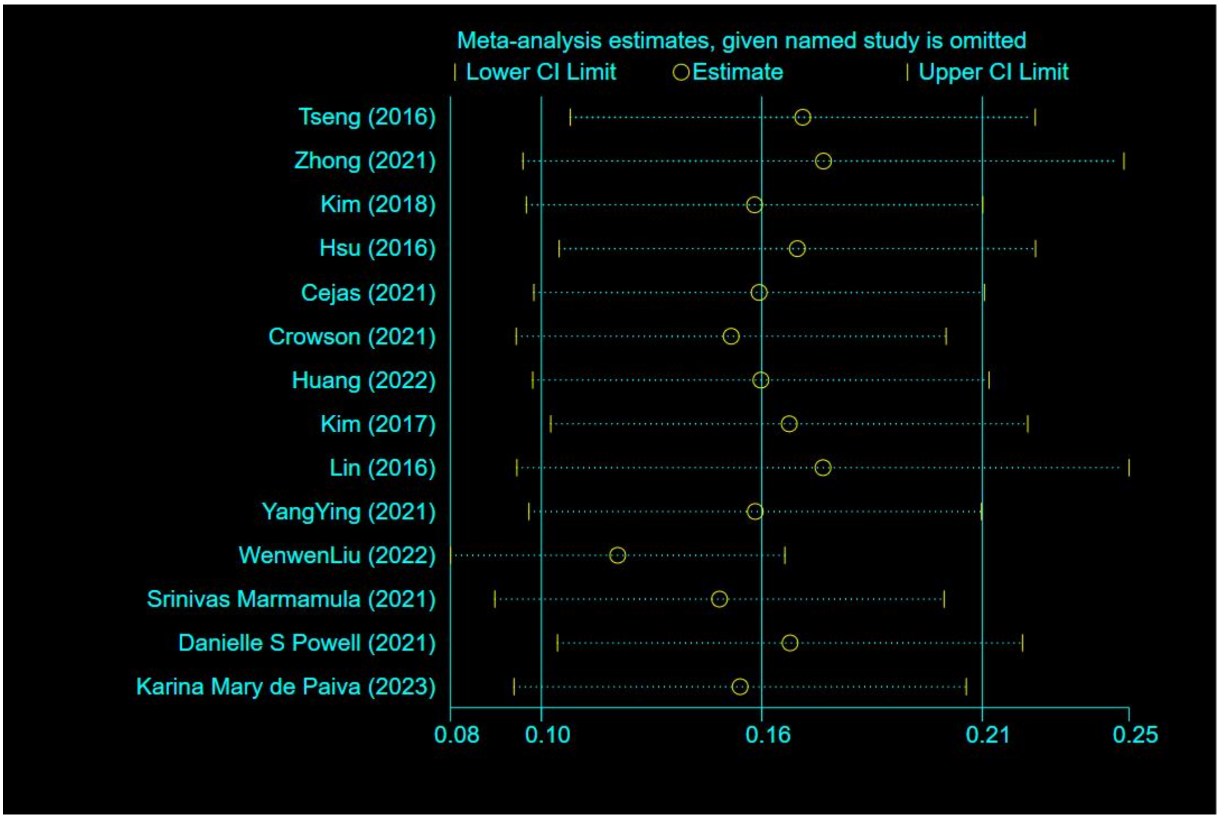

Supplementary Figure 5. Sensitivity analysis of the incidence of depression among patients with sensorineural hearing loss

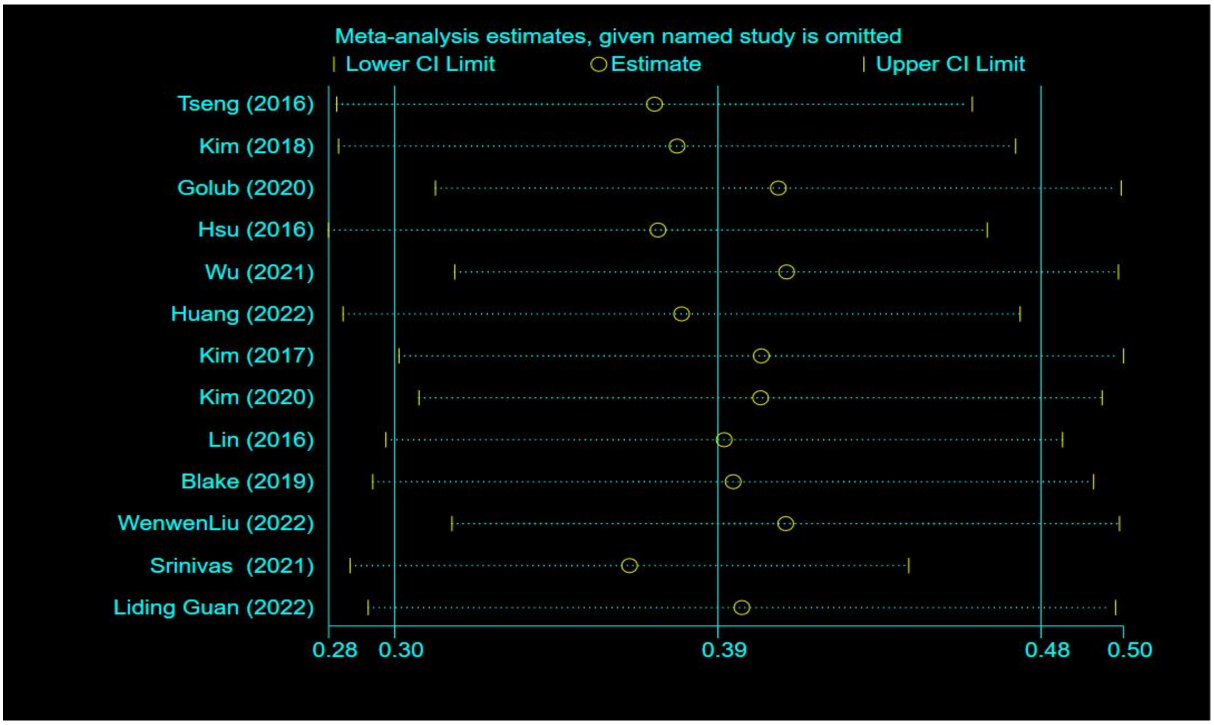

Supplementary Figure 6. Sensitivity analysis of the risk of depression among patients with sensorineural hearing loss

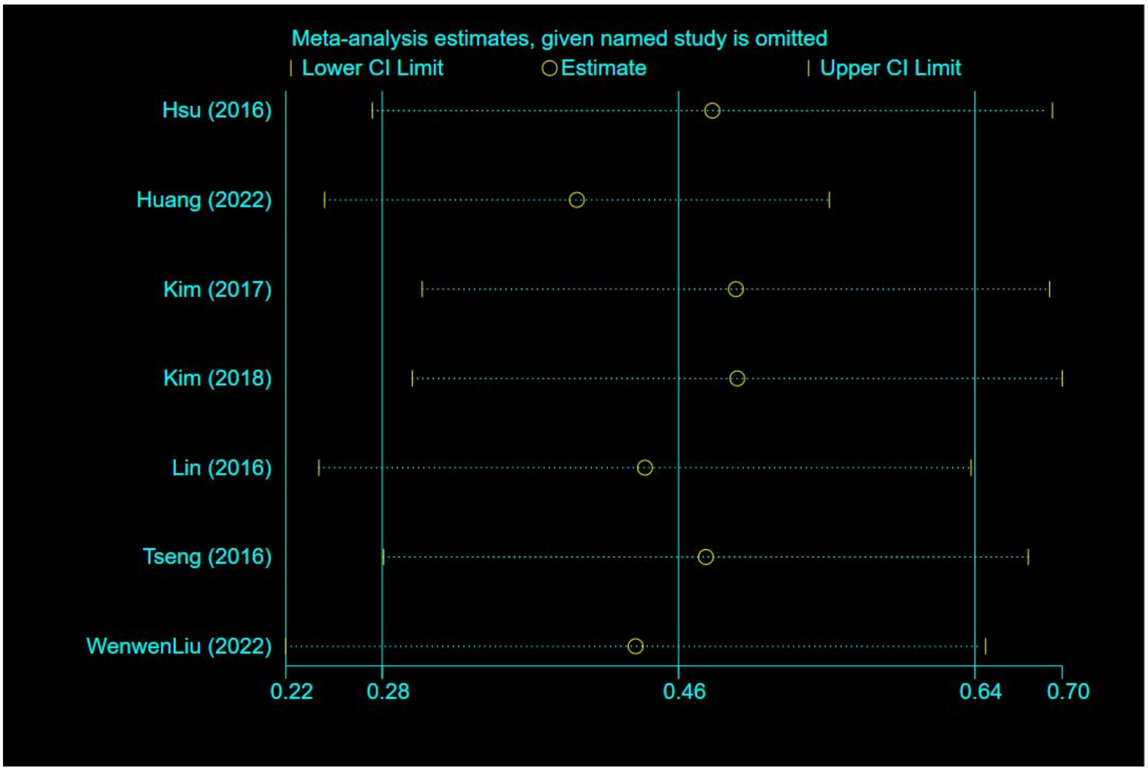

Supplementary Figure 7. Sensitivity analysis of the incidence of sensorineural hearing loss among depressed patients

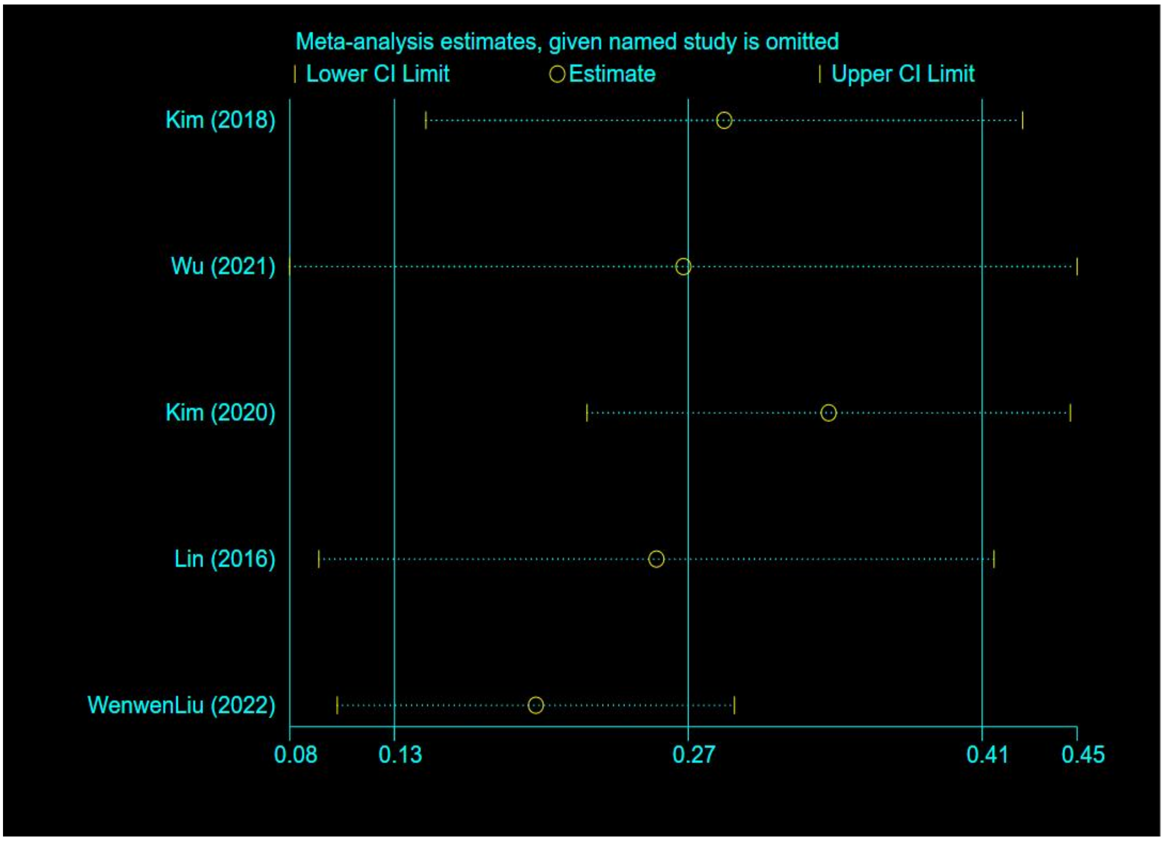

Supplementary Figure 8. Sensitivity analysis of the risk of developing sensorineural hearing loss among depressed patients

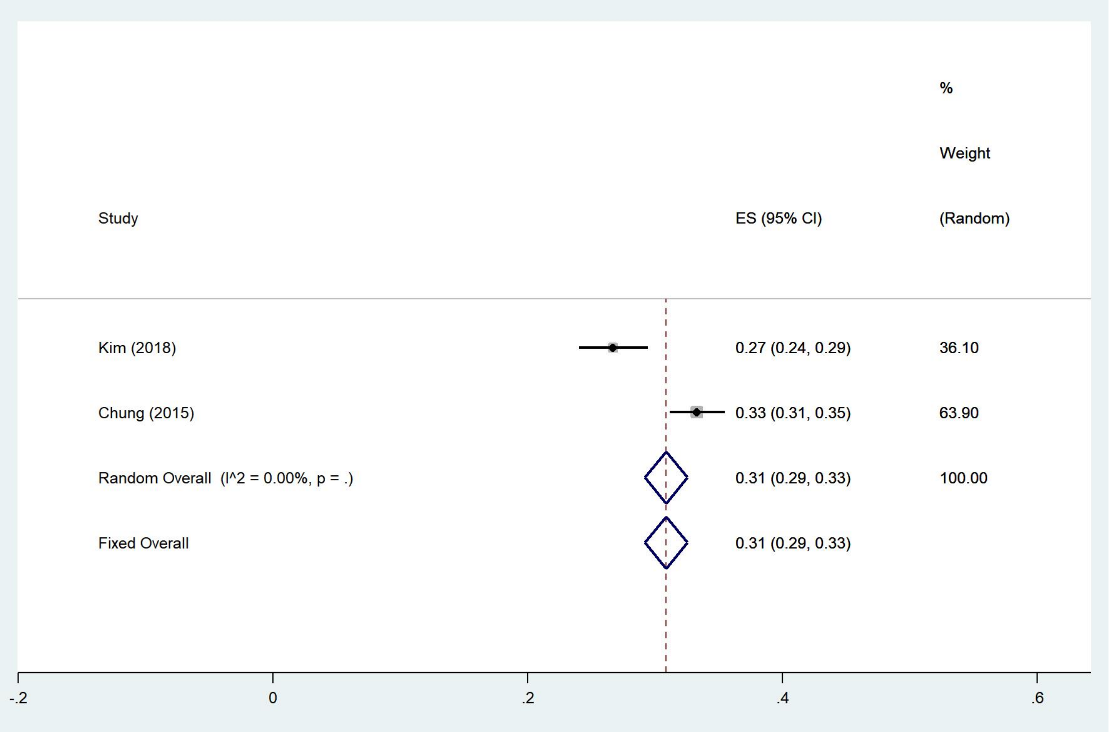

Supplementary Figure 9. Forest plot of prevalence of sensorineural hearing loss among anxiety disorders patient

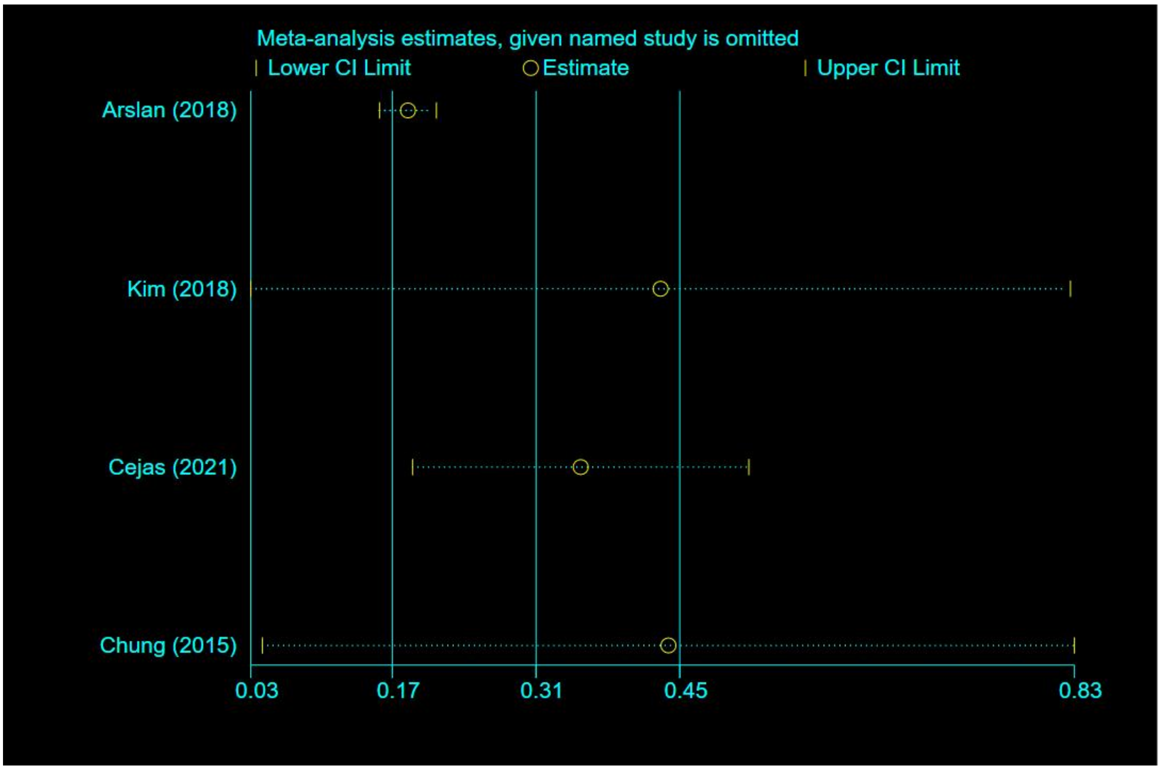

Supplementary Figure 10. Sensitivity analysis of the prevalence of anxiety disorders among patients with sensorineural hearing loss

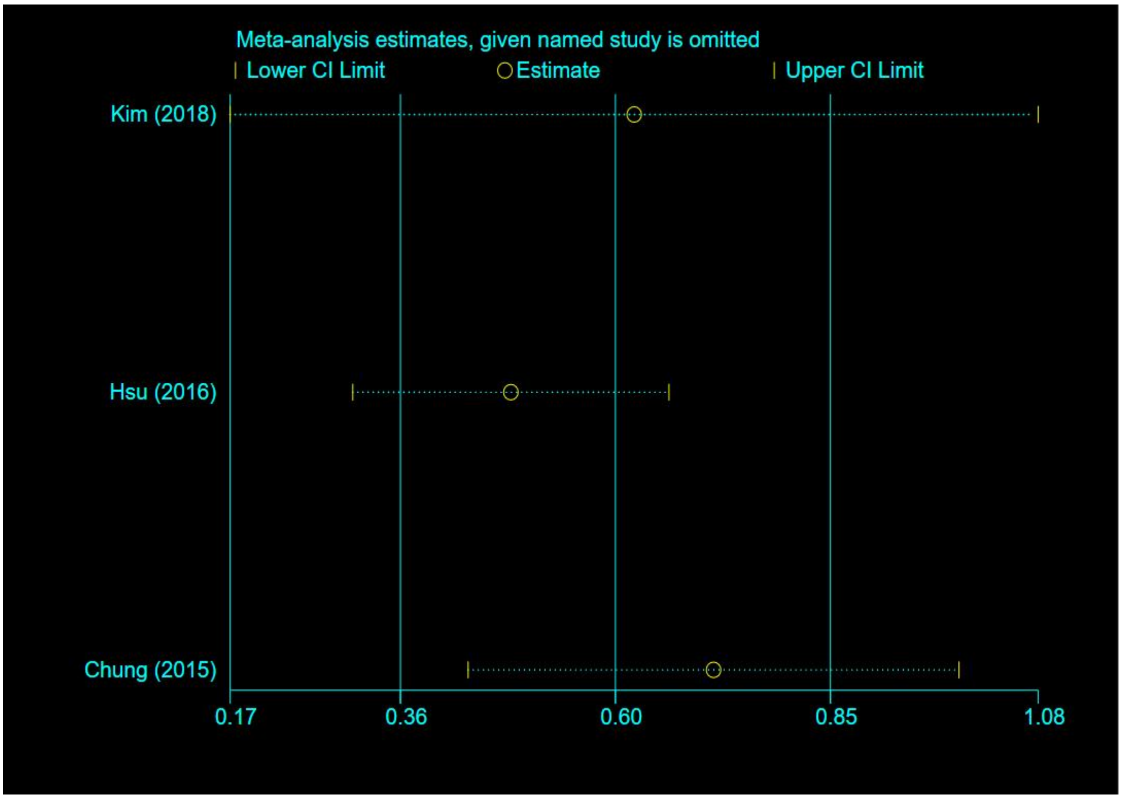

Supplementary Figure 11. Sensitivity analysis of the risk of anxiety disorders among patients with sensorineural hearing loss

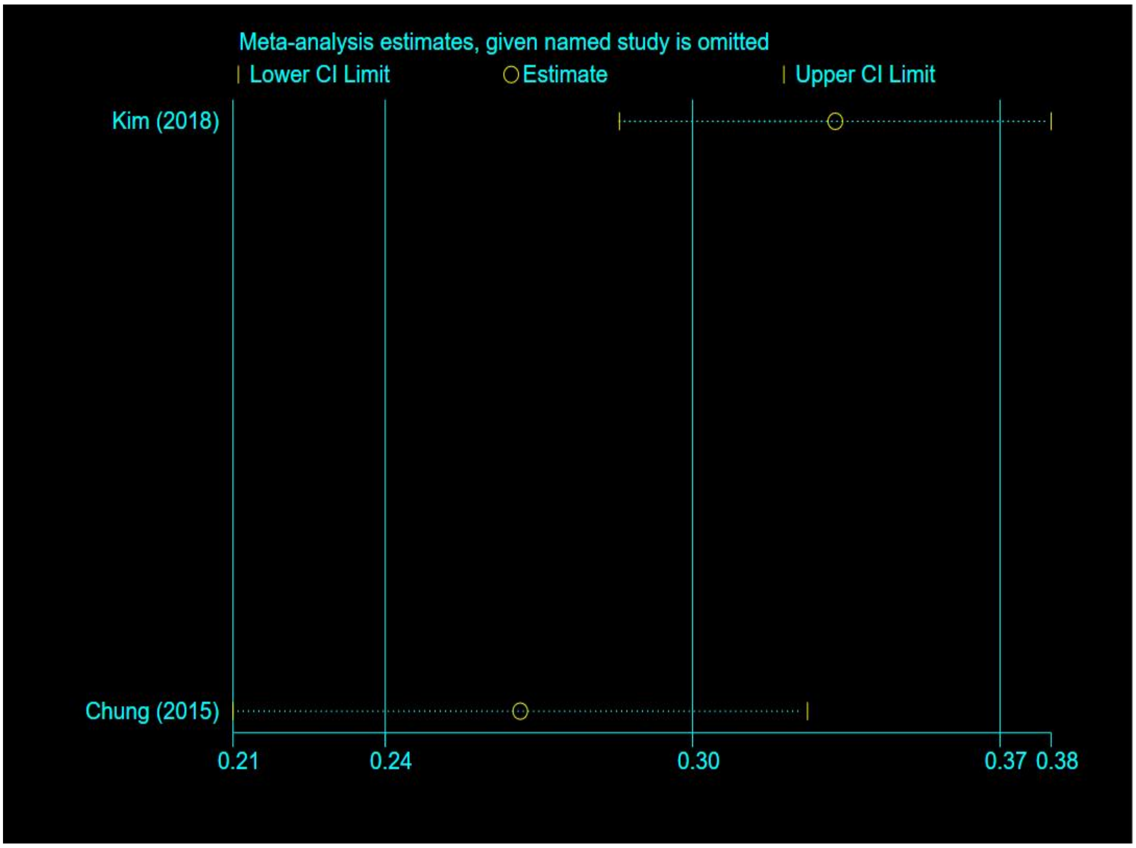

Supplementary Figure 12. Sensitivity analysis of the incidence of sensorineural hearing loss among patients with anxiety disorders
